# Antiosteolytic Bisphosphonate Metallodrug Coordination Networks: Dissolution Profiles and *In Vitro/In Vivo* Toxicity toward Controlled Release

**DOI:** 10.1021/acsabm.5c01890

**Published:** 2025-11-18

**Authors:** Elpiniki Chachlaki, Maria Vassaki, Petri A. Turhanen, Duane Choquesillo-Lazarte, Christina N. Banti, Sotiris K. Hadjikakou, Konstantinos D. Demadis

**Affiliations:** a Crystal Engineering, Growth and Design Laboratory, Department of Chemistry, 150573 University of Crete, Heraklion Crete GR-71003, Greece; b School of Pharmacy, University of Eastern Finland, Biocenter Kuopio, P.O. Box 1627, Kuopio FIN-70211, Finland; c Laboratorio de Estudios Cristalográficos, IACT-CSIC, Granada 18100, Spain; d Laboratory of Biological Inorganic Chemistry, Department of Chemistry, University of Ioannina, Ioannina GR-45110, Greece; e Institute of Materials Science and Computing, University Research Center of Ioannina (URCI), Ioannina GR-45110, Greece

**Keywords:** osteoporosis, bisphosphonates, clodronate, medronate, metal phosphonates, controlled release, magnesium, calcium, strontium

## Abstract

Coordination compounds were synthesized and structurally characterized containing biocompatible alkaline earth metal ions and the bone-seeking agents clodronate (CLOD, (dichloromethanediyl)­bis­(phosphonate)) and medronate (MED, methylenediphosphonate). Dimensionality in these structures ranges from 0D (Mg-CLOD, Ca-CLOD) to 1D (Ca-CLOD-CP) to 2D (Ca-MED, Sr-CLOD). The salt Na_2_–CLOD (used as a reference) and the CLOD coordination compounds with Mg^2+^, Ca^2+^, and Sr^2+^ were utilized as controlled release systems (excipient-containing tablets) of the active drug CLOD in acidic conditions that mimic the human stomach (pH = 1.3). Release of Ca^2+^ ions from the Ca-CLOD system was also monitored. The same experiments were carried out for the MED and Ca-MED systems. The drug release profiles were compared, and it was found that all Mg/Ca/Sr-containing compounds exhibit variable deceleration of the “active” CLOD release compared to the Na-containing reference. The calculated initial rates (μmol CLOD/min) followed the order Na (1.67) > Mg (1.32) > Sr (0.97) > Ca (0.81/0.70). The values were 1.44 and 0.57 for the MED and Ca-MED systems. This behavior was rationalized based on the structural idiosyncrasies of each system. The overall drug release profile for each system was the result of several structural factors, such as H-bonding interactions, strength of the metal–O­(phosphonate) bonds, and packing density, but also crystal morphological/textural factors. These compounds were also tested for their toxicity at the concentration of 100 μM *in vitro* (micronucleus assay) and *in vivo* (brine shrimp *Artemia salina*) and were found to be of low toxicity.

## Graphical abstract

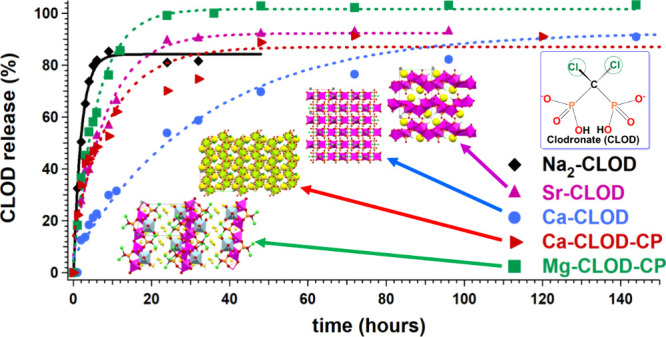

## Introduction

Bisphosphonates (BPs) are organic structural analogs of inorganic pyrophosphate. They have been used as treatments for bone-related conditions (the most well-known is osteoporosis) since 1977.^1^ Conceptually, the O bridge between the P atoms in pyrophosphate is replaced with a C atom. The latter is linked to two more substituents; one of them is usually −OH and the other an organic fragment. The nature of this organic moiety determines several aspects of their action and is the basis for their categorization into generations.^2^ There is an abundance of information in the scientific literature on their synthesis (with several approaches being “green”^3^) and their diverse pharmaceutical action.^4^

Due to their low bioavailability,^5^ most BPs are administered orally (in the form of pills/tablets) containing a rather “high” dose of the active pharmaceutical agent, thus inducing several undesirable side effects. Among the various strategies proposed to reduce these side effects is the fabrication of controlled release systems (CRSs) that could administer the active BP drug in a predictable and controllable fashion. Current approaches include principally dissolution-, or diffusion-controlled systems (reservoir or monolithic systems), and water penetration-systems (osmotic, swelling, chemically controlled). A new approach takes advantage of the strong affinity of BPs for metal ions in aqueous solutions to generate metal-BP “complexes”, which are commonly sparingly soluble and conveniently precipitate out of solution.^6^ This property can be systematically exploited by deliberately constructing metal-containing hybrid materials by combining a BP of choice with a preselected biologically acceptable metal ion, such as some of the alkaline-earth cations Mg^2+^, Ca^2+^, and Sr^2+^. The working hypothesis here is that the incorporation of the BP in the metal-BP hybrid matrix dramatically reduces the solubility of the BP drug, in comparison to its “free” solid form (without the metal cation). Recently, we reported some initial studies on metal-drug coordination polymers with the linkers etidronate, pamidronate, alendronate, neridronate,^7^ risedronate,^8^ and zoledronate.^9^

Clodronate (CLOD) is a “non-nitrogen” BP and is in clinical use under several brand names (Bonefos, Clasteon, Ostac, Loron, Difosfonal, Mebonat, Ossiten). It is one of the few BPs that contain neither a −OH nor an organic fragment on the central carbon atom. Instead, the two substituents are chlorine atoms. It is a highly polar compound and is freely soluble in water, with low lipophilicity. It is a potent inhibitor of osteoclast mediated bone resorption. It has been extensively tested in patients with advanced breast cancer, myelomatosis, hypercalcemia, Paget’s disease, and osteoporosis. The reader is referred to a recent excellent review that analyzes in detail its history as a successful drug.^10^ The structurally similar methylenediphosphonic acid (medronic, MED) is the “simplest” of the BPs (having two H’s on the central carbon atom), usually used in a radiolabeled form as a radiotracer in medical imaging.^11^ Its complex with ^99m^Tc is a pharmaceutical product (with the trade name Mdp-Bracco) used in nuclear medicine (bone scintigraphy) to localize bone metastases as well as other diseases that can alter the natural turnover in the bone.^12^ Figure 1 shows the schematic structures of several BPs currently in use, along with those of CLOD, MED and the archetypal pyrophosphate (PPi).

**Figure 1:**
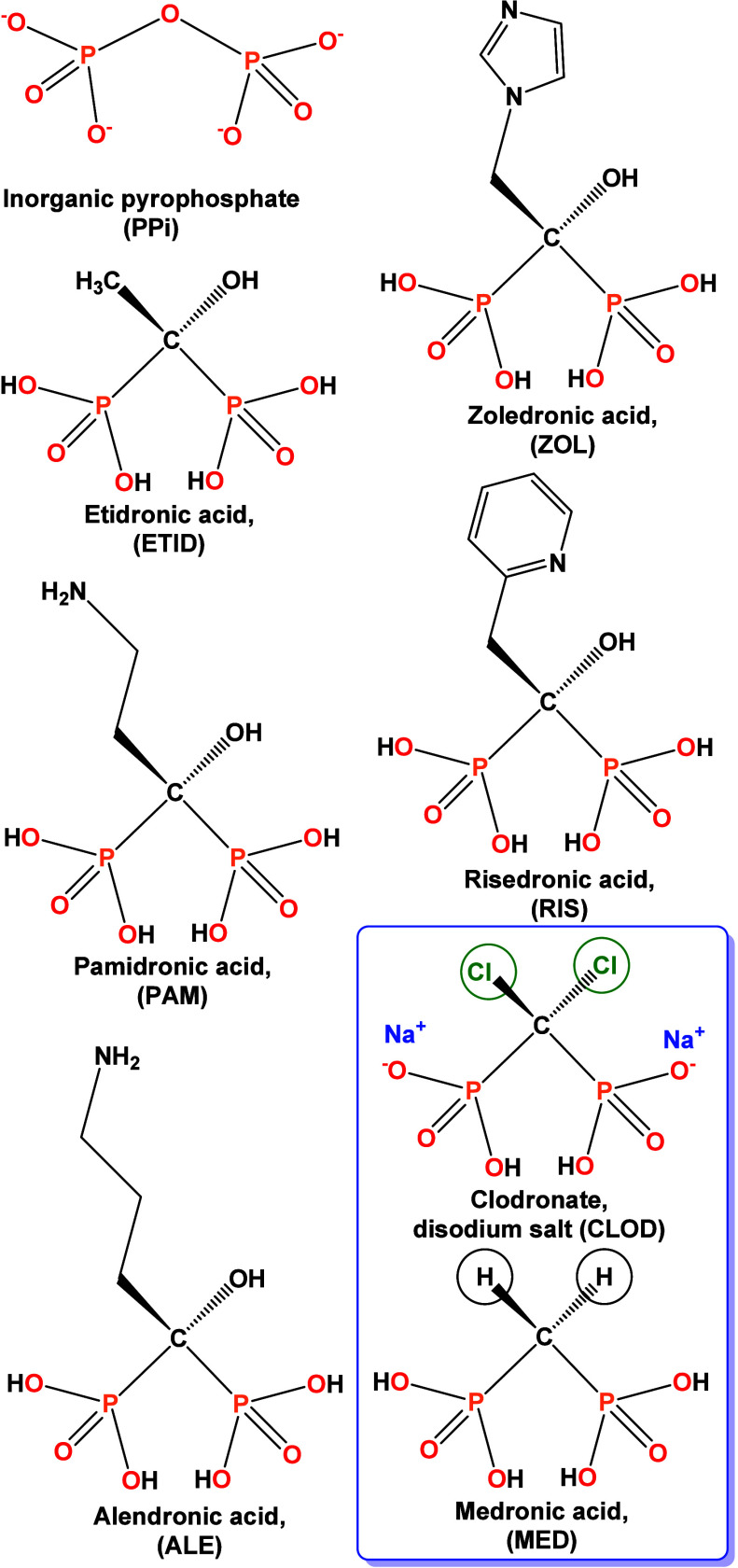
Schematic structures of several BPs currently in clinical use. The compounds CLOD and MED (used in this research) are placed in the frame.

In this paper, we report the bulk synthesis, structural characterization and dissolution profiles of the following precisely defined metal-CLOD compounds: Mg-CLOD-CP (CP stands for “coordination polymer”; it is a 1D network), the dinuclear complex Mg-CLOD-D (D stands for “dimer”, which is actually a byproduct during the synthesis of Mg-CLOD-CP), Ca-CLOD (0D mononuclear complex), Ca-CLOD-CP (1D coordination polymer), and Sr-CLOD (2D coordination polymer). The Ca^2+^ derivative of MED (Ca-MED, a 2D coordination polymer) is also reported for structural comparison reasons. To aid the reader, we show the reported compounds in Scheme 1.

**Scheme 1:**
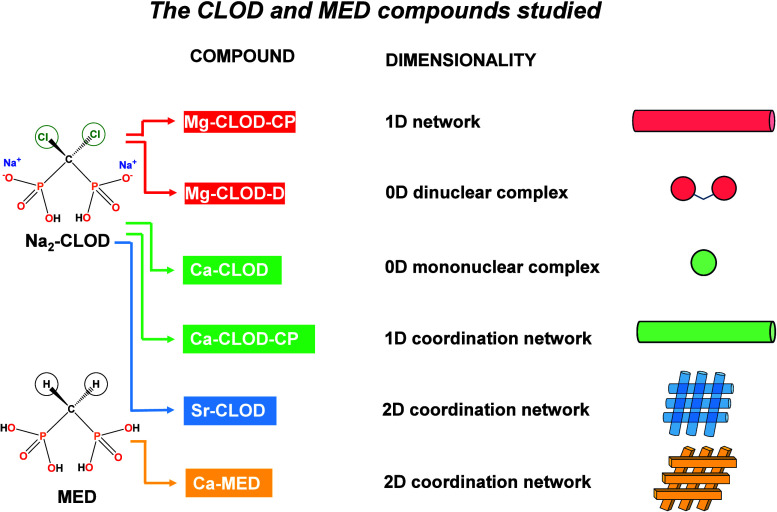
Notation and Structural Dimensionality Coding of the Synthesized, Characterized and Evaluated CLOD and MED Compounds Reported in This Paper

In addition, the dissolution kinetics of the active drug CLOD from fabricated 3-excipient tablets containing each of four metal-CLOD compounds (Mg-CLOD-CP, Ca-CLOD, Ca-CLOD-CP and Sr-CLOD) are studied under acidic conditions (at pH = 1.3, mimicking the human stomach) and are compared to the “free” CLOD system (disodium salt, Na_2_–CLOD). Tablets were used as the preferred and most practical delivery system in order to more closely simulate the common way of drug administration to a patient (pills). For comparison, the release features and kinetics of “free” MED and the Ca-MED compounds from fabricated tablets are also studied. To the best of our knowledge, this is the first systematic controlled release study of the CLOD and MED drugs and their metal derivatives. The selection of the three alkaline earth cations is based on the fact that Mg^2+^ and Ca^2+^ are apparently nontoxic and biocompatible, and Sr^2+^ has well documented health benefits. Although Sr is not considered as an essential element, studies indicate that supplementing a diet with Sr^2+^ may help reduce bone pain, increase bone mineral density, and reduce the risk of certain fractures.^13,14^ Notably, the precise role of Sr^2+^ in the human body is not fully understood.^15^ The BP dissolution systems studied herein combine a high drug loading (e.g., 40–60% CLOD loading), with the simultaneous release of the active drug, together with a beneficial metal ion. No other metal ions (e.g., transition metals) were investigated, because of potential toxicity reasons. Finally, we report detailed *in vitro* (micronucleus assay) and *in vivo* (brine shrimp *Artemia salina*) toxicity studies of these compounds. The present study adds valuable information to the “mosaic” of drug release data on other metal-BP systems studied in our group.^7–9^ Importantly, the quantification of drug release by ^31^P NMR spectroscopy is both reproducible (with low error) and reliable. Based on the controlled release studies originated from our group on other metallodrug systems (with bisphosphonate drugs such as etidronate, pamidronate, alendronate, risedronate, and zolendronate),^7–9^ each system displays its own idiosyncrasies. This is because each drug has its own functional groups (hydroxyl, amino, pyridine, imidazole), besides the common “bisphosphonate” structural feature, while the nature of the metal cation varies (Mg, Ca, Sr, Ba).

## Experimental Section

The research presented in this manuscript does not involve animal/human research.

### Materials

All reagents that were utilized as sources of metal ions were from commercial sources. MgCl_2_·6H_2_O was purchased from Scharlau. CaCl_2_·2H_2_O, Ca­(NO_3_)_2_·4H_2_O and SrCl_2_·6H_2_O were purchased from Sigma-Aldrich. The tablet excipients lactose (Serva), cellulose (Merck) and silica (Alfa-Aesar) were from commercial sources. Deionized (DI) water was used in all experiments and was produced from a laboratory ion exchange column. Deuterium oxide (99.9 atom % D) containing 0.05 wt % sodium 3-(trimethylsilyl)-propionate-2,2,3,3-d4 (TSP) was used for MED quantification via ^1^H NMR and was purchased from Deutero.

### Instrumentation

#### Scanning Electron Microscopy

Elemental analyses and SEM images of the morphology of the metal–BPs collected with a JEOL JSM-6390LV electron microscope.

#### Single Crystal X-ray Diffraction

Measured crystals were prepared under inert conditions immersed in perfluoropolyether as protecting oil for manipulation. Suitable crystals were mounted on MiTeGen Micromounts, and these samples were used for data collection. Data were collected with a Bruker D8 Venture diffractometer with graphite monochromated CuKα radiation (λ = 1.54178 Å). The data were processed with APEX3 suite.^16^ The structures were solved by intrinsic phasing using the ShelXT program,^17^ which revealed the position of all non-hydrogen atoms. These atoms were refined on F^2^ by a full-matrix least-squares procedure, using the anisotropic displacement parameter.^18^ All hydrogen atoms were located in difference Fourier maps and were included as fixed contributions riding on attached atoms with isotropic thermal displacement parameters 1.2- or 1.5-times those of the respective atom. The Olex2 software was used as a graphical interface.^19^ Molecular graphics were generated using the Mercury software.^20^ The coordination geometries of the metal-CLOD compounds were determined using the SHAPE software (Shape Software, 521 Hidden Valley Road, Kingsport, TN 37663 USA, https://www.shapesoftware.com). The crystallographic data for Mg-CLOD-D (CCDC number 2340861), Ca-CLOD-CP (CCDC number 2369174), and Ca-MED (CCDC number 2410009) were deposited with the Cambridge Crystallographic Data Center (Structural data (Table S1) and the cif files are provided in the Supporting Information). The structures of Mg-CLOD-CP,^21^ Ca-CLOD,^22^ and Sr-CLOD^22^ (of single crystals obtained via the gel method) have been published elsewhere.

#### Powder X-ray Diffraction

The powder X-ray diffraction (XRD) patterns were performed on PANalytical X’Pert Pro diffractometer, a configuration of the Bragg–Brentano, equipped with monochromator Ge(111) (Cu Κ_α1_) and detector X’Celerator. All compounds reported showed good agreement between the calculated and measured XRD patterns.

#### Other Instrumentation

Attenuated Total Reflectance Infrared (ATR-IR) spectra were recorded with a FT/IR-4200 JASCO Spectrophotometer, equipped with PIKe ATR (MIRacle), DTGS detector, Ge crystal plate. These experiments were set at a resolution of 4 cm^–1^ in the range of 4000–600 cm^–1^. NMR spectra (^1^H, ^31^P­{^1^H} and ^13^C­{^1^H}) were recorded on a Bruker DPX-300 spectrometer in D_2_O. The solvent residual peak was used as a standard for ^1^H NMR measurements in D_2_O (4.79 ppm) and in ^13^C NMR measurements CD_3_OD was added as a reference (49.00 ppm). H_3_PO_4_ (85% aqueous solution) was used as an external standard in the ^31^P NMR measurements. Thermogravimetric analysis (TGA) data were recorded on an SDT-Q600 analyzer from TA Instruments. The temperature varied from room temperature (RT) to 900 °C at a heating rate of 10 °C·min^–1^ under air or N_2_ flow. Elemental analyses (C, H, N) were measured on a TruSpec Macro CHN-S analyzer.

### Synthetic Protocols

#### Synthesis of [(Dichloro-phosphono-methyl)­phosphonic Acid, Disodium Salt, Tetrahydrate], Clodronate Disodium Tetrahydrate (Na_2_[Cl_2_C­(PO_3_H)_2_]·4H_2_O, Coded as “Na_2_–CLOD”)

Na_2_–CLOD was synthesized by following the method previously reported, but with some modifications.^23^ Below, the synthesis protocol is provided. Methylenebis­(phosphonic acid) tetraisopropyl ester (50 g, 0.15 mol) was added by small portions to a cooled solution (0–5 °C) of 12% NaOCl (600 mL) and NaHCO_3_ (101 g, 1.2 mol) with vigorous stirring for over 1–1.5 h. Afterward, the reaction mixture was first stirred at 0–5 °C for 0.5 h and then at ambient temperature for 2 h. The reaction mixture was diluted with water (1000 mL) and extracted twice with CH_2_Cl_2_ (250 mL). The combined CH_2_Cl_2_ layers were washed with water (250 mL), dried with MgSO_4_ and then the CH_2_Cl_2_ was evaporated to dryness *in vacuo*. The residue was dissolved in conc. HCl (300 mL) and refluxed for 2 h before evaporation *in vacuo*. The residue was dissolved in MeOH (200 mL) and re-evaporated *in vacuo* before the residue was dissolved in water (100 mL) and pH adjusted to 4.5–5 with 50% NaOH. EtOH (400 mL) was added by stirring (maintained for 2 h) and then the precipitate was filtered, washed with 50% EtOH/water and finally with pure EtOH and dried under reduced pressure. The final product was obtained as white powder (36.5 g, 87% yield). ^13^C­{^1^H} NMR (D_2_O, CD_3_OD as reference) δ 77.7 (t, ^1^J_CP_ = 128.7, P–C–P). ^31^P­{^1^H} NMR δ 8.8 ppm (D_2_O, pH not adjusted). Peak assignments can be found in Figures S1 and S2 in the SI. NMR data were consistent with those reported in the literature.^23^ Finally, thermogravimetric analysis was conducted in order to quantify the exact number of water molecules after the isolation of the product. The mass reduction (20%) at 100 °C corresponds to the four water molecules, (Figure S3, Supporting Information).

#### Synthesis of Methylenediphosphonic Acid (CH_2_(PO_3_H_2_)_2_ Coded as “MED”)

MED was synthesized according to a published procedure.^24^

#### Synthesis of {[Mg­(H_2_O)_6_]­[Mg­(Cl_2_C­(PO_3_)_2_)­(H_2_O)]·7H_2_O}_n_ (Coded as “Mg-CLOD-CP”)

Na_2_–CLOD (58 mg, 0.2 mmol) and MgCl_2_·6H_2_O (81.2 mg, 0.4 mmol) were dissolved in ∼ 10 mL DI H_2_O under stirring until fully dissolved. The solution pH was adjusted to 7.0 (using stock solutions of NaOH as needed). The final mixture was left under quiescent conditions for solvent evaporation. After 10–15 days (depending on the occasional ambient temperature) a colorless crystalline product formed, it was isolated by filtration, rinsed with DI water and left to dry under air. Bulk product purity was confirmed by powder X-ray diffraction (comparison of the calculated and experimental powder patterns, see Figure S4, Supporting Information), so no CHN analyses were performed. Yield: 57 mg (53%). Syntheses at lower pH values were also tested, but the Mg-CLOD-D appeared as an impurity (see below).

#### Synthesis of Mg_2_[Cl_2_C­(PO_3_)_2_(H_2_O)_7_]·5H_2_O (Coded as “Mg-CLOD-D”)

This compound was not possible to be synthesized as a single phase and was always observed as a byproduct during the synthesis of Mg-CLOD-CP when carried out at pH values <6. Otherwise, the stoichiometric amounts were the same as in the synthesis of Mg-CLOD-CP. Elemental analysis (%) on manually selected crystals: Calculated for CH_26_Cl_2_Mg_2_O_19_P_2_, M.W. 523.68: C 2.29, H 4.96; Found: C 2.17, H 5.06.

#### Synthesis of Ca­[Cl_2_C­(PO_3_H)_2_(H_2_O)_5_] (Coded as “Ca-CLOD”)

Na_2_–CLOD (28.8 mg, 0.1 mmol) and CaCl_2_·2H_2_O (29.4 mg, 0.2 mmol) were dissolved in ∼ 10 mL DI H_2_O under stirring until fully dissolved. The solution pH was adjusted to 2.1 (using stock solutions of HCl as needed). The final mixture was left under quiescent conditions for solvent evaporation. After 7–10 days (depending on the occasional ambient temperature) a colorless crystalline product formed, it was isolated by filtration, rinsed with DI water and left to dry under air. Bulk product purity was confirmed by powder X-ray diffraction (comparison of the calculated and experimental powder patterns, see Figure S5, Supporting Information), so no CHN analyses were performed. Yield: 17 mg (46%).

#### Synthesis of {Ca_2_[Cl_2_C­(PO_3_)_2_(H_2_O)_6_]·H_2_O}_n_ (Coded as “Ca-CLOD-CP”)

Na_2_–CLOD (28.9 mg, 0.1 mmol) and CaCl_2_·2H_2_O (14.7 mg, 0.1 mmol) were dissolved in ∼ 20 mL DI H_2_O under stirring until fully dissolved. The solution pH was adjusted to 6 (using stock solutions of NaOH as needed). The final mixture was left under quiescent conditions for solvent evaporation. After 3–4 days (depending on the occasional ambient temperature) a colorless crystalline product formed. It was isolated by filtration, rinsed with DI water and left to dry under air. Bulk product purity was confirmed by powder X-ray diffraction (comparison of the calculated and experimental powder patterns, see Figure S6, Supporting Information). Yield: 11 mg (50%). Calculated for CH_14_Ca_2_Cl_2_O_13_P_2_, M.W. 449.20: C 2.67, H 3.16; Found: C 2.75, H 3.32.

#### Synthesis of {Sr_2_[Cl_2_C­(PO_3_)_2_(H_2_O)_4_]·H_2_O}_n_ (Coded as “Sr-CLOD”)

Na_2_–CLOD (36 mg, 0.1 mmol) and SrCl_2_·6H_2_O (27 mg, 0.1 mmol) were dissolved in ∼ 10 mL DI H_2_O under stirring until fully dissolved. The solution pH was adjusted to 5.5 (using stock solutions of NaOH and HCl, as needed). The final mixture was left under quiescent conditions for solvent evaporation. After 7–10 days (depending on the occasional ambient temperature) a colorless crystalline product formed, it was isolated by filtration, rinsed with DI water, and left to dry under air. Bulk product purity was confirmed by powder X-ray diffraction (comparison of the calculated and experimental powder patterns, see Figure S7, Supporting Information), so no CHN analyses were performed. Yield: 28 mg (55%).

#### Synthesis of {Ca­[H_2_C­(PO_3_H)_2_(H_2_O)]·H_2_O}_n_ (Coded as “Ca-MED”)

MED acid (9 mg, 0.05 mmol) and Ca­(NO_3_)_2_·4H_2_O (12 mg, 0.05 mmol) were dissolved in ∼ 10 mL DI H_2_O under stirring until fully dissolved. The solution pH was adjusted to 4 (using stock solutions of NaOH and HNO_3_, as needed). The final mixture was left under quiescent conditions for solvent evaporation. After 10–15 days (depending on the occasional ambient temperature) a colorless crystalline product formed, it was isolated by filtration, rinsed with DI water, and left to dry under air. Bulk product purity was confirmed by powder X-ray diffraction (comparison of the calculated and experimental powder patterns, see Figure S8, Supporting Information). Elemental analysis (%): Calculated for CH_8_CaO_8_P_2_, M.W. 252.10: C 4.80, H 3.22; Found: C 4.75, H 3.26.

### Fabrication of Tablets for the Release of BPs from CLOD- and MED-Containing Compounds

Tablets were prepared by mechanical mixing of ground powders (with a mortar-and-pestle) of the drug component (850 μmol of CLOD content) and three commonly used excipients, i.e., lactose, cellulose, and silica. Subsequently, a tablet was prepared by applying 10 tons of pressure in a hydraulic press. The tablet’s total weight was 1.000 g. Identical tablets that contained equimolar CLOD or MED amounts of the metal–CLOD (Na^+^, Mg^2+^, Ca^2+^, Sr^2+^) and Ca-MED compounds, and the three excipients were fabricated. MED could not be quantified by ^1^H NMR in 3-excipient tablets because of the methylene proton peak overlap with lactose. Hence, 2-excipient tablets were prepared in the same manner that contained only cellulose and silica, *sans* lactose. The total weight of each tablet was kept constant at 1.000 g. The quantities used in the tablets are shown in Table 1.

**Table 1:** Quantities of Active Agents (Metal–CLOD, Metal = Na^+^, Mg^2+^, Ca^2+^, Sr^2+^, MED, and Ca-MED) and Excipients Utilized for Tablet Preparation

**Tablet**	**Na_2_–CLOD**	**Mg–CLOD-CP**	**Ca–CLOD**	**Ca-CLOD-CP**	**Sr–CLOD**	**MED** ^a^	**Ca-MED**
MW (g/mol)	360.92	541.68	373.03	447.12	506.18	176.00	252.10
Drug (g)	0.307	0.461	0.317	0.380	0.430	0.150	0.213
Lactose (g)	0.231	0.180	0.228	0.207	0.190	-^b^	-^b^
Cellulose (g)	0.231	0.180	0.228	0.207	0.190	0.425	0.394
Silica (g)	0.231	0.180	0.228	0.207	0.190	0.425	0.394

a Used as “free acid”.

b No lactose was used for pellet fabrication because of MED −CH_2_– peak interference in the ^1^H NMR spectra.

### Quantification of Released CLOD

Each tablet (prepared as mentioned above) was submerged in a glass beaker filled with 50 mL of deionized water whose pH was adjusted to 1.3 by using hydrochloric acid. It is understood that this medium does not represent or mimic a real gastrointestinal environment, which is far more complex, involving enzymatic activity, dynamic pH variations, and interactions with biomolecules. We purposely selected a “simpler” solution medium and focused only on the low pH as the determinant of the release, avoiding other factors that are certainly important, but may complicate results interpretation. The drug-containing tablet was placed in a plastic net and was immersed into the solution just above the stirring bar. Mild stirring was applied to ensure solution homogeneity. Aliquots of the solution were withdrawn (sample volume 350 μL) and the sampling pattern was hourly for the first 6 h, then every 3 h until the 12th hour, and finally every 12 h until the 48th hour of the release experiment. After the 48th hour, samples were withdrawn every 24 h or every 48 h or longer, if necessary. The experiment was stopped when three consecutive measurements gave the same value (plateau). Each aliquot was placed in a standard quartz NMR tube, and then the standard solution (150 μL) was added. The standard solution was prepared by dissolving chromium­(III) acetylacetonate (10 μmol) and potassium dihydrogen phosphate (0.6 μmol) in D_2_O (6 mL). The 150 μL of the standard solution contained 15 μmol potassium dihydrogen phosphate. Quantification of CLOD concentration in each sample was achieved by peak integration (singlet at 8.31 ppm, attributed to the P of the two phosphonate groups) in the ^31^P­{^1^H} NMR spectrum and comparing it to the peak of the KH_2_PO_4_ (singlet at 0.008 ppm) standard solution peak [-PO_4_]. For a representative ^31^P NMR spectrum, see Figure S9, Supporting Information. Initial rates were calculated based on the initial linear portion of the curve. All release experiments were carried out at ambient temperature.

### Quantification of Released MED

The same procedure described above was used for MED quantification, with the difference that the sample from the working solution was mixed with D_2_O TSP standard solution (150 μL). The concentration of the D_2_O TSP standard solution was 4.337 μmol. Quantification of MED concentration in each sample was achieved by peak integration (−CH_2_−) in the ^1^Η NMR spectrum and its comparison against the [-Si­(CH_3_)_3_] TSP peak. Initial rates were calculated based on the initial linear portion of the curve at the early release stages. All release experiments were carried out at ambient temperature. The excellent reproducibility, accuracy and reliability of this methodology was thoroughly assessed and discussed previously.^5–7,24^

### Quantification of Released Ca^2+^

The EDTA titration method was used, and the experimental procedure is described in the SI. The Ca-CLOD was studied for Ca^2+^ release, as a representative system.

### Biological Evaluation

The biological experiments, including cell viability assessment using the SRB (Sulforhodamine B) assay and micronucleus evaluation, were carried out in DMSO/DMEM solutions containing 1% v/v DMSO. Stock solutions of the compounds (0.01 M) were freshly prepared in DMSO and subsequently diluted with the culture medium (DMEM) to achieve the desired final concentrations. The SRB Assay and micronucleus evaluation were performed as described in detail previously.^25–27,27,29^ The evaluation of the *in vivo* toxicity with the brine shrimp (*Artemia salina*) assay was performed as previously reported.^30^

## Results

### Synthesis and Characterization of the Mg/Ca/Sr-CLOD and Ca-MED Compounds

The crystallization of the compounds Mg-CLOD-CP (1D), Ca-CLOD (0D) and Sr-CLOD (2D) was previously performed in silicate gels.^21,22^

However, for the drug release studies, bulk quantities of pure products were required, therefore, reproducible bulk syntheses of these compounds in pure form were necessary. This proved to be a challenge, but eventually it was successful by exploring experimental variables in synthetic efforts (reactant stoichiometries and pH values). The synthesis pH is an important parameter for the reaction outcome because it determines the protonation state of the ligand. The Ca-CLOD complex was synthesized in crystalline form at pH 2.1, where the CLOD ligand is doubly deprotonated. The 1D coordination polymer Mg-CLOD-CP was synthesized at pH ∼ 7 and the CLOD ligand is fully deprotonated with a “-4” charge. Since the Mg:CLOD ratio in the linear chain stoichiometry is 1:1, the polymer is anionic (with a “-2” charge per building unit, which is charge-balanced by a Mg­(H_2_O)_6_^2+^ cation). Not unexpectedly, the Ca-CLOD-CP compound incorporates a fully deprotonated CLOD^4–^ linker due to the “higher” synthesis pH of 6.0. The CLOD^4–^ linker requires two Ca^2+^ cations for charge balance, yielding a neutral monodimensional framework. The 2D coordination polymer Sr–CLOD was synthesized in mildly acidic pH (5.5). The solution pH is a crucial determinant for isolating pure and tractable products. In general, excessively low pH will cause either no reaction, or crystallization of unreacted ligand, whereas high pH will result in fast product precipitation that is usually amorphous (causing characterization problems, e.g., *via* powder XRD), or with low crystallinity. Inevitably, extensive experimentation with various solution pH values must be carried out. The crystalline solids of all compounds were isolated and studied by scanning electron microscopy as well. The synthetic scheme, size, morphology and texture of the crystals are shown in Figure 2.

**Figure 2:**
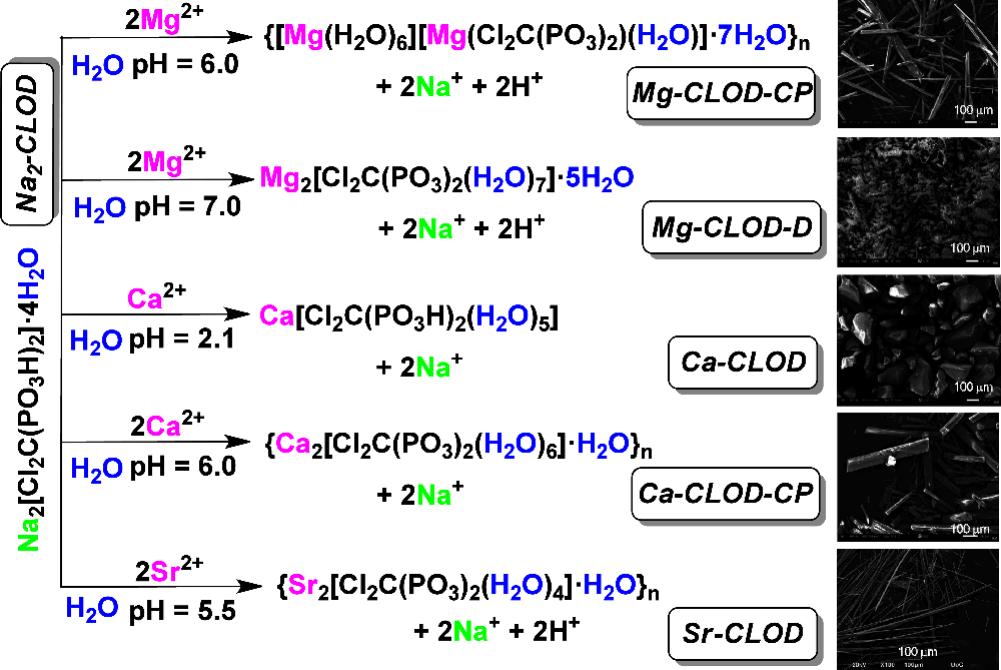
Reaction scheme for the synthesis of the metal-CLOD compounds and SEM images of their single crystals.

As will be discussed below in the description of the structures, the CLOD ligand is found either doubly, or quadruply deprotonated, depending on the synthesis pH. When the negative charge is fully balanced by the M^2+^ cations, neutral compounds (Mg-CLOD-D, Ca-CLOD, Ca-CLOD-CP, and Sr-CLOD) are produced. In the case of the anionic 1D polymer Mg-CLOD-CP the negative charge is balanced by a Mg­(H_2_O)_6_^2+^ cation per structural unit. Full deprotonation of bisphosphonates occurs at fairly high pH regimes. For example, the p*K*_a_ values for etidronic acid (hydroxyethylidene-1,1-diphosphonic acid, also a first generation drug) are 1.8, 2.8, 7.0 and 11.2,^31^ which means that the last two removable protons are dissociated at pH > 7. Apparently, this is not the case with CLOD, whose p*K*_a_ values are much lower, 1.7, 2.1, 5.7 and 8.3.^32^ Although the synthesis pH values seem to be insufficiently high for full deprotonation of CLOD, complexation to the M^2+^ facilitates the removal of the third and fourth proton, leading to full ligand deprotonation. We have observed this during our research, particularly with lanthanide ions, which cause unexpected phosphonate ligand deprotonation at pH values as low as 1.0.^33^

The CLOD compounds (Na_2_–CLOD, Mg-CLOD-CP, Ca-CLOD, Ca-CLOD-CP, and Sr–CLOD) were studied by ATR-IR spectroscopy (see Figure S10, Supporting Information). The vibrational frequencies between 2900 and 3600 cm^–1^ are attributed to O–H antisymmetric stretching vibration of the water molecules (either in the lattice or metal-coordinated. They present variability between the four compounds due to their different type (lattice and coordinated waters) and environment. The bands in the region 1550–1720 cm^–1^ are assigned to the water bending modes. The spectral region 900–1200 cm^–1^ is complex and includes several characteristic vibrations related to the –PO_3_ moieties of CLOD.^34^ The bands in the regions 745–763 cm^–1^ and 865–900 cm^–1^ are assigned to the asymmetric and symmetric C–Cl vibrations of CLOD and are present in all compounds.^35^ Vibrational spectroscopy is a rapid, reliable, and valuable diagnostic tool to characterize metal phosphonate products and to differentiate them from the starting materials.

The published p*K*_a_ values for MED acid are 2.19, 3.26, 7.00, and 9.97.^36^ Hence, it is expected that at pH ∼ 4 (during the synthesis of Ca-MED) the ligand is doubly deprotonated, with a charge of “-2”. Hence, the Ca^2+^ cation fully balances the negative charge, and the product Ca-MED is a neutral framework. Ca-MED shows several vibrations in the fingerprint region assigned to the phosphonate groups (see Figure S10, Supporting Information), which are distinct from those of MED acid.

### Structural Description of the Metallodrugs

#### Mg-CLOD-D

The structural features of the compound are shown in Figure 3. The coordination environment of each Mg center is octahedral, according to SHAPE. Each Mg center is coordinated by three terminal water molecules (O4, O5, O6) in a *mer* fashion. Two phosphonate oxygens (O1, O2) occupy the other two coordination sites, while the sixth ligand is a bridging water molecule (O7). The Mg–O (O being a terminal water or a phosphonate oxygen) are around 2 Å and are found in the expected range.^37^ The Mg–O bond distance with the bridging water molecule is surprisingly long, 2.425 Å. There are six lattice water molecules per dimeric unit, which create a complicated network of hydrogen bonds. The ligand exists in its tetrakis-deprotonated state (CLOD^4–^) due to the “higher” crystallization pH of 7.0.

**Figure 3:**
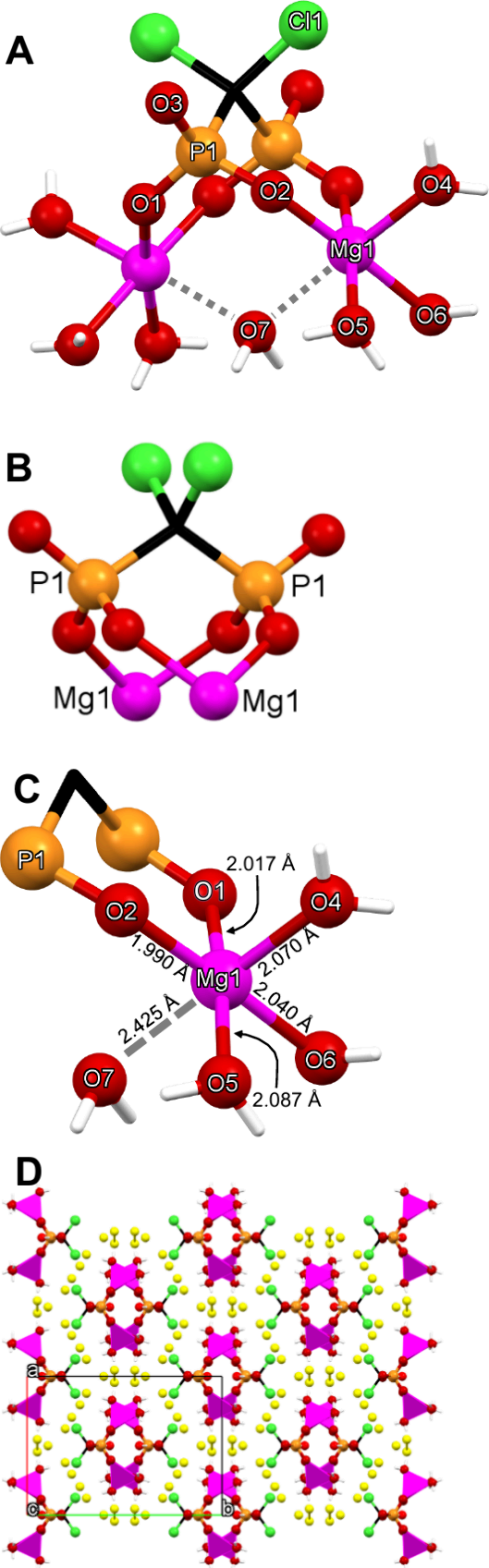
(A) Structure of the Mg dimer, showing the numbering scheme. (B) The doubly bridging mode of the CLOD^4–^ ligand. (C) Mg–O bond distances. (D) Packing of the structure along the *c*-axis. Color codes: Mg, magenta; P, orange; O, red; C, black; Cl, green; H, white. The lattice water molecules are highlighted in yellow.

#### Mg-CLOD-CP

The structural features of the compound are shown in Figure 4. There are three types of Mg centers. The first is the Mg(3) in the hexa-aqua octahedral complex Mg­(H_2_O)_6_^2+^. Its metric features are identical to previously reported material containing this cation.^38^ The other two, Mg(1) and Mg(2) belong to the 1D chain, and their coordination environment is also octahedral, according to SHAPE. Although Mg(1) and Mg(2) demonstrate identical coordination environments, they are crystallographically unique because the two types of CLOD^4–^ anions (with phosphorus atoms P1 and P2) participate in slightly different hydrogen bonding schemes. Only Mg(1) will be briefly described. It is coordinated by four phosphonate oxygens (2 × O11, and 2 × O21) which occupy the four basal coordination sites, originating from two chelating CLOD ligands (in the tetrakis-deprotonated CLOD^4–^ state due to the synthesis pH of 6.0), while the remaining ligands are the bridging water molecules (O1 and O2), found in a *trans* position. The Mg–O bond distances with the bridging water molecules are fairly long, 2.204 Å and 2.308 Å, but shorter than the corresponding length in Mg-CLOD-D (see above). The are seven lattice water molecules per dimeric unit, which create a complicated network of hydrogen bonds. The Mg–O­(phosphonate) bond distances are found in the expected range (Figure 4).^37^

**Figure 4:**
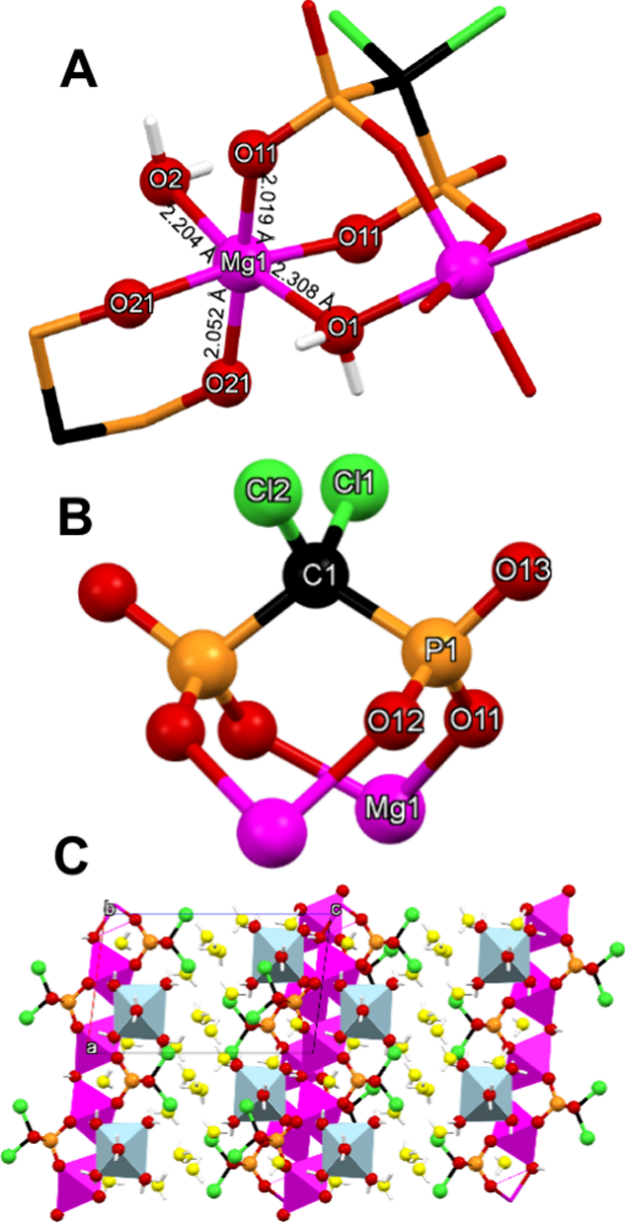
(A) Structure of the dimeric building block in the Mg-CLOD-CP coordination polymer, showing the numbering scheme, with Mg–O bond distances. (B) The doubly bridging mode of the CLOD^4–^ ligand. (C) Packing of the structure along the *b*-axis. Color codes: Mg, magenta; P, orange; O, red; C, black; Cl, green; H, white. The charge-balancing Mg­(H_2_O)_6_^2+^ cations are shown as light blue octahedra. The lattice water molecules are highlighted in yellow.

#### Ca-CLOD

This compound is a mononuclear complex and its structural features are shown in Figure 5. The coordination environment of the 7-coordinated Ca center is a capped octahedron, according to SHAPE. The ligand is found in its bis-deprotonated state (CLOD^2–^, because of the low synthesis pH = 2.1), and it acts as a bidentate chelate, with two of its oxygens (one from each P) coordinating the Ca center. The remaining five ligands are water molecules. Interestingly, there are no lattice water molecules in the structure. All intermolecular hydrogen bonds in the structure form between the phosphonate groups and the coordinated water molecules. The Ca–O bond distances (either Ca-water or Ca-phosphonate) fall within a range observed in other Ca-phosphonates.^39^

**Figure 5:**
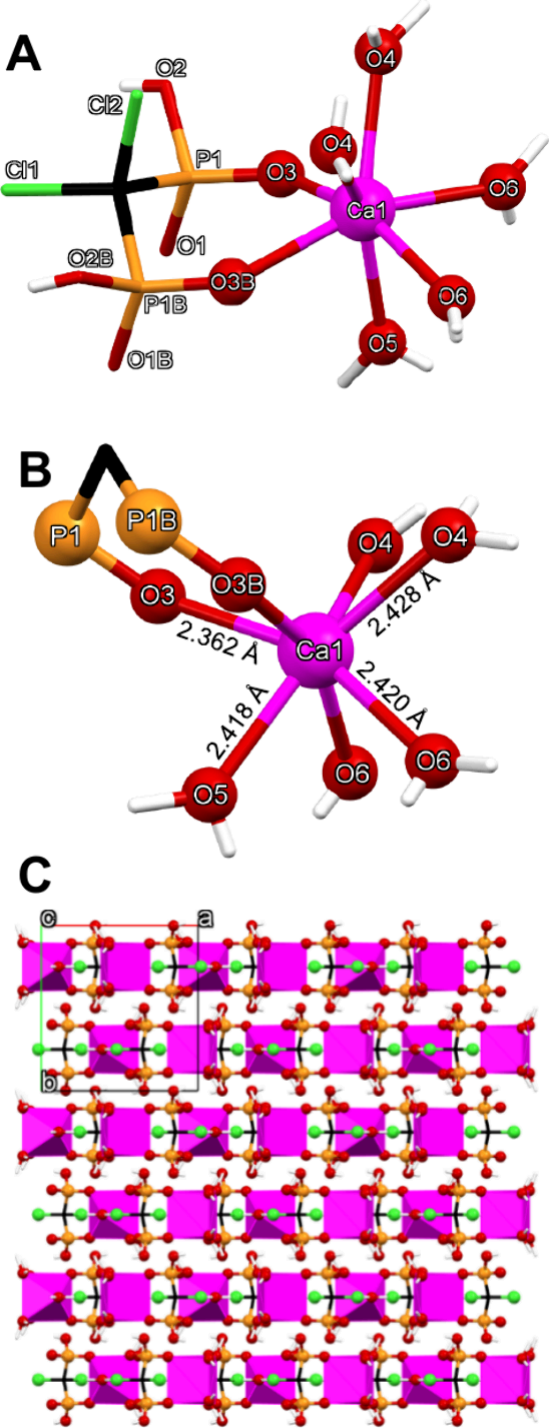
(A) Structure of the Ca-CLOD complex, showing the numbering scheme. (B) Ca–O bond distances. (C) Packing of the structure along the *c*-axis. Color codes: Ca, magenta; P, orange; O, red; C, black; Cl, green; H, white.

#### Sr-CLOD

The structure of Sr-CLOD can be described as a 2D coordination polymer. A representation of the coordination environment of the CLOD ligand (in its tetrakis-deprotonated CLOD^4–^ state due to the synthesis of 5.5) is shown in Figure 6. Each CLOD ligand coordinates to six Sr^2+^ centers via the phosphonate oxygens and one of the Cl substituents. Specifically, the P1 phosphonate binds to one Sr in a terminal fashion (via O3), to two Sr centers in a bridging fashion (*via* O1), while the phosphoryl (P=O_2_) group remains uncoordinated. In the second P2 phosphonate group all oxygens are bridging two Sr centers each. There is also a weak Sr–Cl interaction (3.290 Å). CLOD forms a total of four chelating rings with the Sr centers (one 4-, and three 5-membered rings).

**Figure 6:**
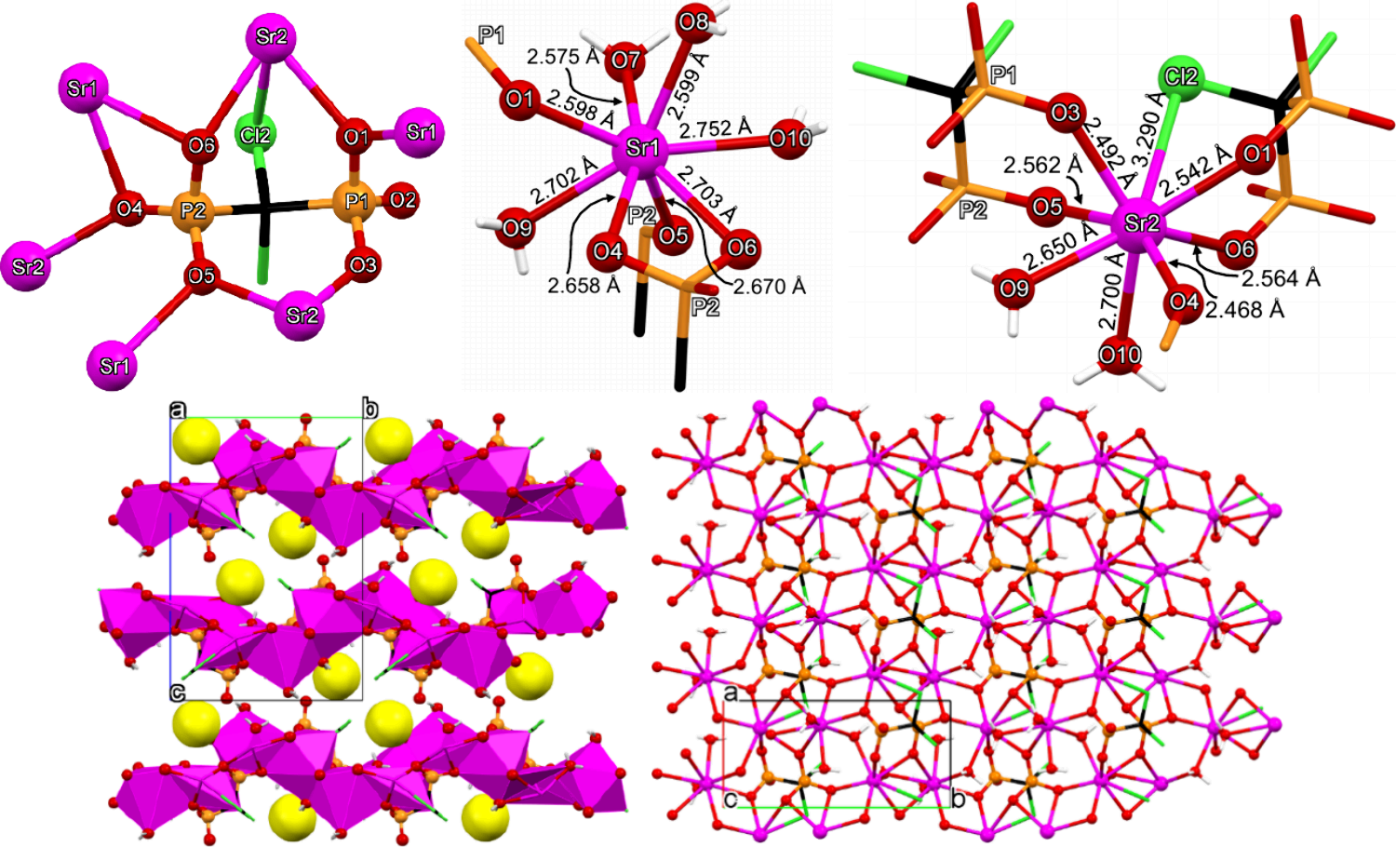
(Upper left) Depiction of the environment of the CLOD tetra-anionic ligand in the structure of Sr–CLOD. The coordination environments of the Sr1 (upper middle) and Sr2 (upper right) centers in the structure of Sr–CLOD. Packing of three 2D layers along the *a*-axis (lower left). The interlayer lattice waters are shown as exaggerated yellow spheres. View of one layer along the *c*-axis (lower right). Color codes: Sr, magenta; P, orange; O, red; C, black; Cl, green; H, white.

All P–O bond lengths are found in a narrow range (1.514–1.529 Å), as a result of complete deprotonation and coordination to the Sr centers. There are two Sr^2+^ centers in the structure of Sr-CLOD. Both are 8-coordinated, but present differences in their specific coordination environment.

Τhe coordination sphere of the Sr1 center can be described as a biaugmented trigonal prism (based on SHAPE analysis), see Figure 6 (upper left). Sr1 is heavily hydrated, as four of the eight coordinating entities are water molecules. The Sr–O­(H_2_O) bond distances are found in the range 2.575–2.752 Å. Two phosphonate oxygens (from different CLOD ligands) coordinate to Sr in a terminal fashion, with bond distances Sr1–O1­(P1) 2.598 Å and Sr1–O5­(P2) 2.670 Å. The remaining two sites on Sr1 are occupied by two oxygens (O4 and O6 from phosphonate P2) that form the 4-membered chelating ring mentioned above. The bond distances are Sr1–O4 2.658 Å and Sr1–O6 2.703 Å.

Τhe coordination sphere of the Sr2 center can be described as a triangular dodecahedron (based on SHAPE analysis), see Figure 6 (upper right). There are two water molecules coordinated to Sr2 in a *cis* arrangement, with bond distances Sr2–O9 2.650 Å and Sr2–O10 2.700 Å. Two CLOD tetra-anions are coordinated to Sr2, in distinctly different fashion. One forms a 5-membered chelating ring via two oxygens (O3 and O5), but from different PO_3_ groups, with bond distances Sr2–O3 2.492 Å and Sr2–O5 2.562 Å. The other CLOD ligand coordinates as the first, but, additionally with one Cl atom forming a Sr–Cl bond, 3.290 Å. Hence, this CLOD ligand is tris-chelating. Sr2 is bound by two oxygens (O1 and O6), but from different PO_3_ groups, with bond distances Sr2–O1 2.542 Å and Sr2–O6 2.564 Å.

Sr-CLOD is a 2D layered compound, Figure 6 (lower left and right). Propagation of the Sr-CLOD molecular unit within each layer is achieved *via* the extended bridging ability of the tetra-anionic ligand CLOD. The intralayer space incorporates “sandwiched” lattice water molecules, that interact with the upper and lower layers *via* hydrogen bonding interactions with phosphonate oxygens. Specifically, there is one lattice water molecule (O11) per asymmetric unit in the structure of Sr-CLOD, Figure 6 (lower left). It is situated in the interlayer space, and it forms three hydrogen bonds, one with a phosphonate oxygen (O2) from a layer “above” (O···O 2.708 Å) and two with two O atoms (O9 and O10) from two Sr2-coordinated water molecules (both on Sr2) (O2···O9 2.972 Å and O2···O10 2.824 Å). All Sr–O bond distances are within the expected range.^8,9,40–42^

#### Ca-CLOD-CP

The structure of Ca-CLOD-CP can be described as a 1D coordination polymer. A representation of the coordination environment of the CLOD ligand (in its tetrakis-deprotonated CLOD^4–^ state due to the synthesis of 6.0) is shown in Figure 7. Each CLOD ligand coordinates to four Ca^2+^ centers and utilizes all its phosphonate oxygens, see Figure 7, upper left. Each phosphonate group binds to two Ca centers and one of the O’s acts in a bridging fashion. CLOD forms a total of four chelating rings with the Ca centers (two 3-, and two 6-membered rings).

**Figure 7:**
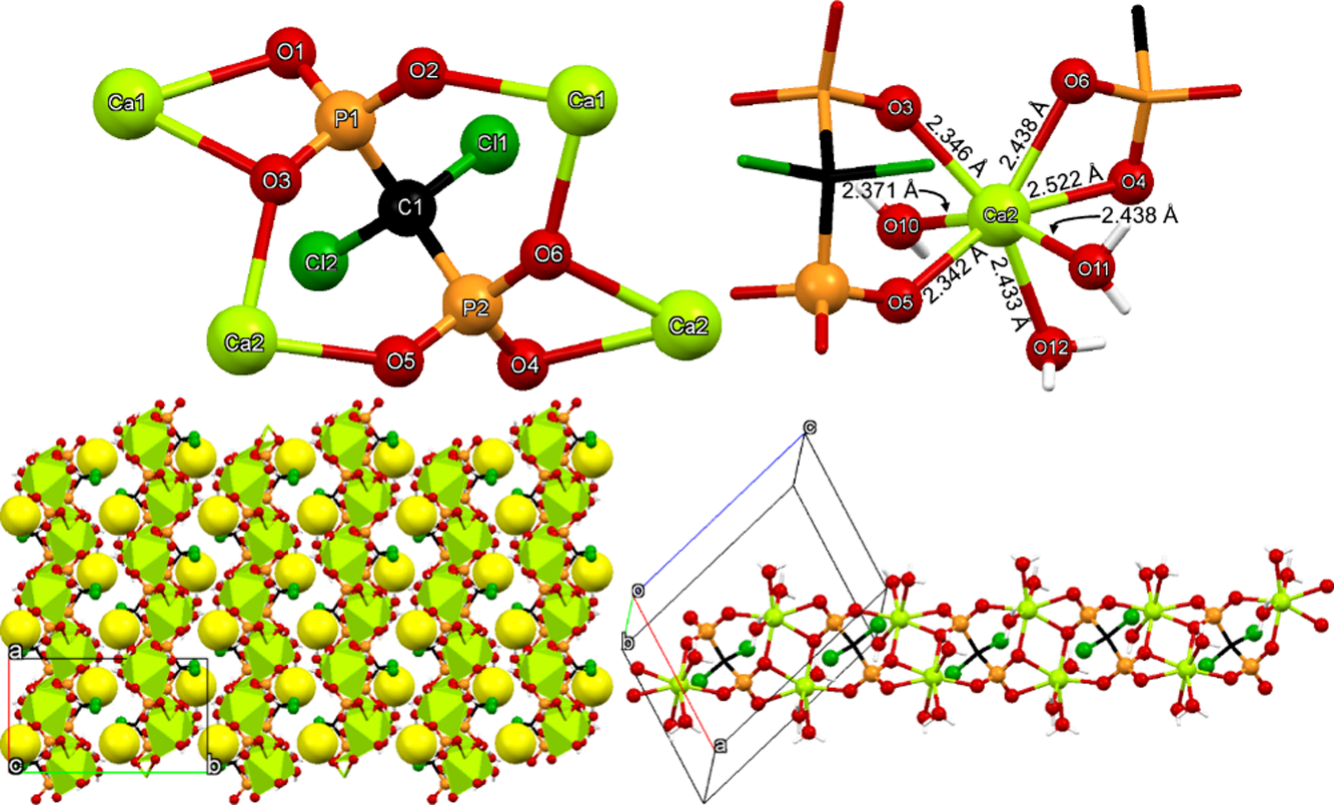
(Upper left) Depiction of the environment of the CLOD tetra-anionic ligand in the structure of Ca–CLOD-CP. The coordination environment of Ca2 (upper right). Packing of six 1D chains along the *b*-axis (lower left). The interlayer lattice waters are shown as exaggerated yellow spheres. View of a single 1D chain. Color codes: Ca, light green; P, orange; O, red; C, black; Cl, dark green; H, white.

Although there are two crystallographically distinct Ca centers, their coordination environment is identical. Τhe coordination sphere of the Ca2 center can be described as a capped trigonal prism (based on SHAPE analysis), see Figure 7 (upper right). There are three water molecules coordinated to Ca2 in a *mer* arrangement, with bond distances Ca2–O10 2.371 Å, Ca2–O11 2.438 Å, and Ca2–O12 2.433 Å. Two CLOD tetra-anion is coordinated to Ca2, in distinctly different fashion. One forms a 6-membered chelating ring via two oxygens (O3 and O5), but from different PO_3_ groups, with bond distances Ca2–O3 2.346 Å and Ca2–O5 2.342 Å. A neighboring CLOD ligand uses two O’s from only one phosphonate group and creates a 4-membered ring with Ca2, with bond distances Ca2–O4 2.522 Å and Ca2–O6 2.438 Å.

Ca-CLOD-CP is a 1D chain-type compound, Figure 7 (lower left and right). Propagation of the Ca-CLOD molecular unit within each chain is achieved *via* the extended chelating/bridging ability of the tetra-anionic ligand CLOD. The interchain space incorporates the lattice water molecules, that interact with the neighboring chains *via* hydrogen bonding interactions involving a Ca-coordinated phosphonate O (O···O contact 2.814 Å), and two Ca-bound water molecules (O···O contacts 2.818 Å and 3.036 Å).

#### Ca-MED

The structure of Ca-MED can be described as a 2D coordination polymer. A representation of the coordination environment of the MED^2–^ ligand is shown in Figure 8. Each MED ligand coordinates to four Ca^2+^ centers *via* the phosphonate oxygens, see Figure 8 upper left. Specifically, the P1 phosphonate binds to two Ca centers in a bridging fashion *via* O1 and to two more *via* O1, while the P–OH group remains uncoordinated. The second P2 phosphonate group binds to two Ca centers in a terminal fashion, while the P–OH group remains uncoordinated. MED forms a total of two chelating rings with the Ca centers (one 3-, and one 6-membered rings).

**Figure 8:**
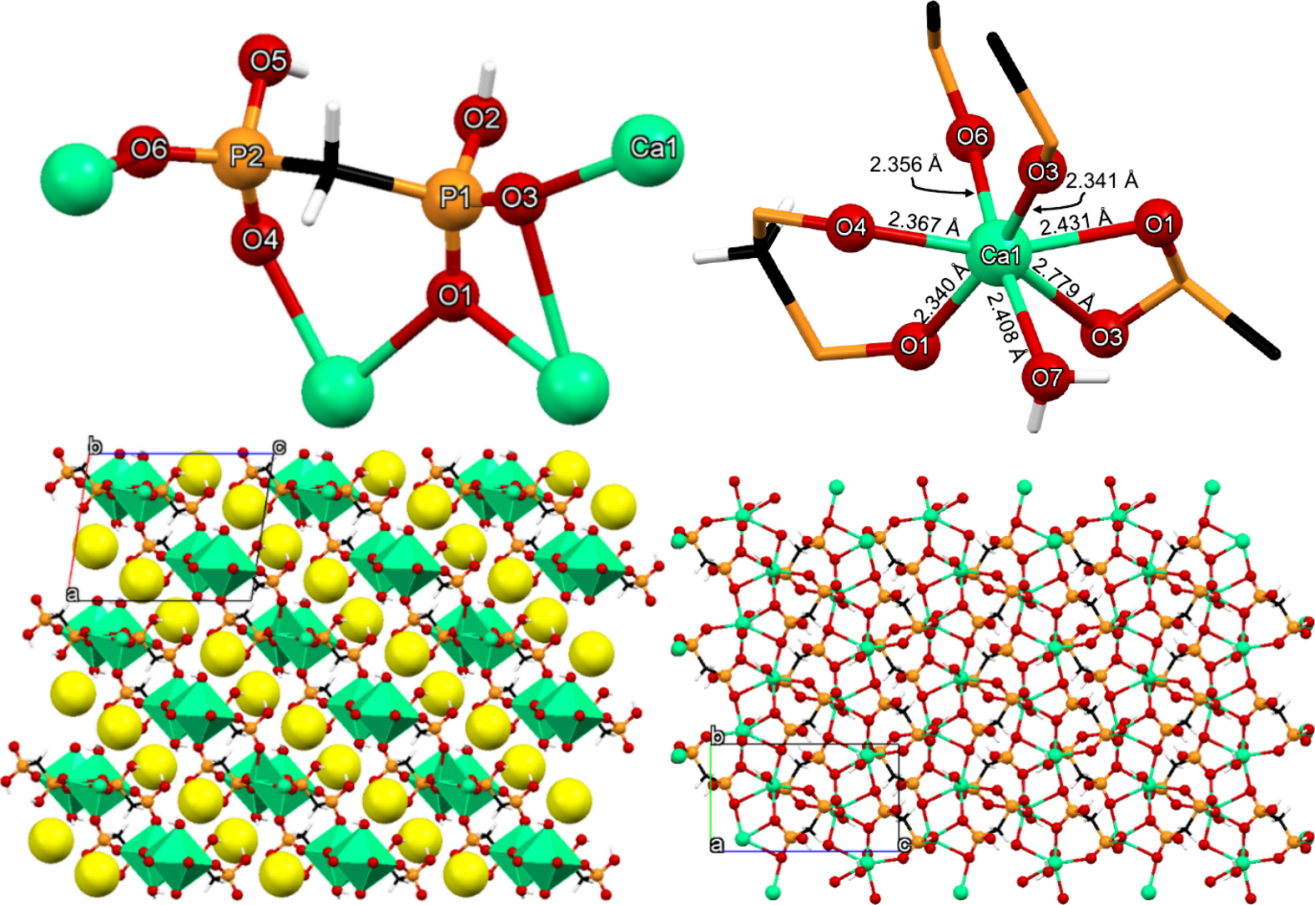
(Upper left) Depiction of the environment of the MED dianionic ligand in the structure of Ca–MED. The coordination environments of the Ca center (upper right). Packing of the 2D layers across the ac diagonal (lower left). The interlayer lattice waters are shown as exaggerated yellow spheres. View of one layer along the *a*-axis (lower right). Color codes: Ca, green; P, orange; O, red; C, black; Cl, green; H, white.

Τhe coordination sphere of the Ca center can be described as a pentagonal bipyramid (based on SHAPE analysis), see Figure 8 (upper right). There is one water molecule coordinated to Ca, with a Ca–O bond distance of 2.408 Å. Four MED ligands are coordinated to each Ca, in distinctly different fashion. Two are terminal (O3 and O6) with bond distances Ca–O3 2.341 Å and Ca–O6 2.356 Å. One MED creates a 4-membered chelating ring with Os from the same phosphonate group, with bond distances Ca–O1 2.431 Å and Ca–O3 2.779 Å. Lastly, one MED creates a 6-membered ring with one O from each phosphonate, with bond distances Ca–O1 2.340 Å and Ca–O4 2.367 Å.

Ca-MED is a 2D layered compound, Figure 8 (lower left and right). Propagation of the Ca-MED molecular unit within each layer is achieved *via* the extended bridging ability of the dianionic ligand MED. The interlayer space incorporates the lattice water molecules, that interact with the upper and lower layers *via* hydrogen bonding interactions with uncoordinated P–OH moieties. Specifically, there is one lattice water molecule (O8) per asymmetric unit in the structure of Ca-MED. It is situated in the interlayer space, and it forms four hydrogen bonds, with the range of O···O interactions found between 2.616 Å and 2.767 Å. The metric features of the MED backbone are comparable to those found in the structure of the “free” MED acid.^43^ All Ca–O bond distances are within the expected range.^39,40,42^

A list of all types of interactions in the structures of Na_2_–CLOD, Mg-CLOD-CP, Ca-CLOD, Sr-CLOD, MED acid and Ca-MED can be found in Table 2. These data will be useful later in the paper for the discussion of the dissolution/controlled release studies.

**Table 2:** All Types of Interactions Around a Single Drug Molecule in the Structures of Na_2_–CLOD, Mg-CLOD-CP, Ca-CLOD, Sr–CLOD, MED, and Ca-MED

**Compound**	**Phosphonate H-bonds (P_A_)**	**Phosphonate H-bonds (P_B_)**	**Cl/H-bonds**	**total H-bonds** ^a^	**M-O(Cl) bonds (PO_3_/Cl)** ^b^	**total interactions**	**lattice H**_**2**_ **O** ^c^	**M^+/2+^ cations**
**Na**_**2**_ **–CLOD**	4	1	0	5	7/1	13	4	2
**Mg-CLOD-CP**	9	9	3	21	4/0	25	7	1^d^
**Ca-CLOD**	6	6	0	12	2/0	19	0	1
**Ca-CLOD-CP**	4	3	1	8	8/0	16	1	2
**Sr-CLOD**	5	3	2	10	9/1	20	1	2
**MED**	5	4	-	9	-	9	0	0
**Ca-MED**	2	4	-	6	6/0	12	1	1

a Only the intermolecular H-bonds are considered.

b The Cl is the substituent on the central C of CLOD.

c Not counting the metal-bridging H_2_O molecules.

d Not counting the [Mg­(H_2_O)_6_]^2+^ countercation.

### Controlled Release Studies of BPs from the CLOD- and MED-Containing Systems

The release curves for all systems are shown in Figure 9 (for CLOD) and Figure 10 (for MED), and some kinetic parameters are collected in Table 3. The four CLOD controlled release systems (CRSs) were evaluated in the form of tablets. In each case, the CLOD-containing compound was mixed and ground with the appropriate excipients in the solid form, and these powders were pressed into tablets. These tablets were immersed into acidic solutions and aliquots were withdrawn at specific time intervals. The detailed protocols for tablet preparation, sampling and drug quantification in solution are described in detail in the Experimental Section. Under the acidic conditions of the drug release experiments, metal–O bond hydrolysis occurs, leading to the degradation of the crystal lattice and the release of CLOD into the acidic medium. The drug release was quantified by ^31^P NMR spectroscopy. In previous studies in our group ^1^H NMR spectroscopy was used for drug quantification,^7–9,24^ but in the case of CLOD there are no NMR-measurable protons in the molecule, so we resorted to ^31^P NMR. These results were plotted in graphs as “% CLOD released” vs time (in hours).

**Figure 9:**
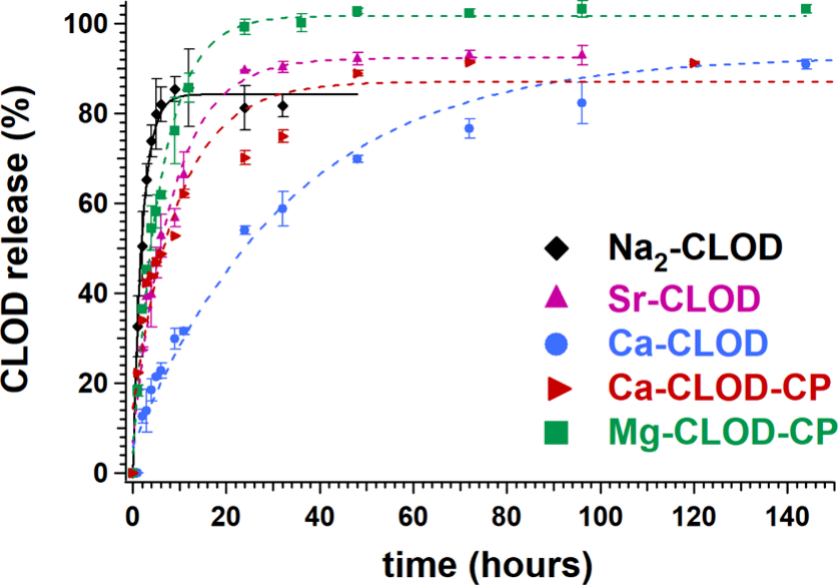
Drug release curves from CLOD-containing tablets (with Na, Mg, Ca, Sr).

**Figure 10:**
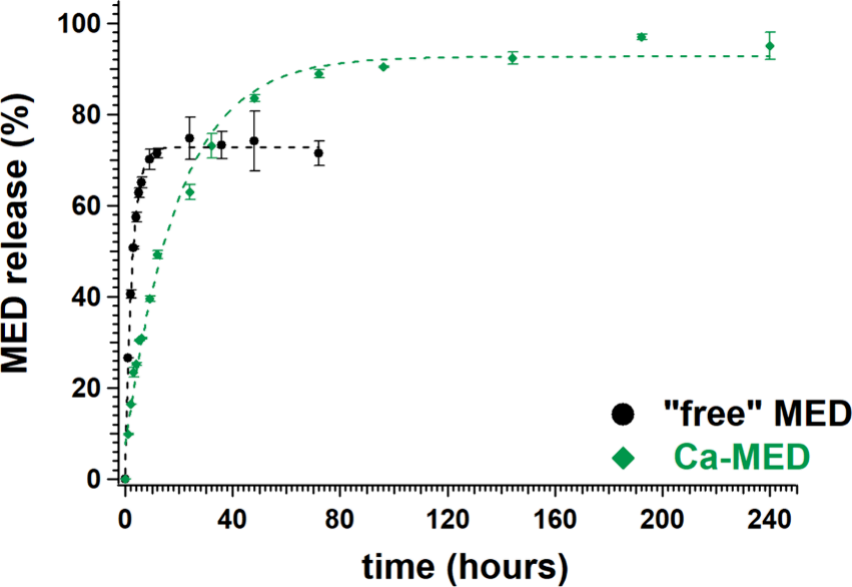
Drug release curves from MED-containing tablets (as acid and with Ca^2+^).

**Table 3:** Kinetic Data for the Drug Release from CLOD- and MED-Containing Tablets

**Metallodrug**	**Initial rate** **(μmol/min)** ^a^	**plateau BP (%)**	***t***_***p***_ **(hours)^b^**	*t*_**1/2**_ **(hours)^c^**
**Na**_**2**_ **–CLOD**	1.67	83	8.5	1.4
**Mg-CLOD-CP**	1.32	100	25.0	3.9
**Ca-CLOD**	0.70	94	144.2	21.6
**Ca-CLOD-CP**	0.81	86	41.33	4.6
**Sr-CLOD**	0.97	90	29.9	4.9
**MED**	1.44	72	11.3	1.7
**Ca-MED**	0.57	91	77.6	11.5

a Calculated based on the initial linear portion of the curve.

b t_p_ is defined as the time required for the plateau value to be reached.

c *t*_1/2_ is defined as the time required for half of the plateau value to be reached.

^1^H NMR peak quantification was successfully applied in the MED and Ca-MED systems (Figure 10 and Table 3). However, the exclusion of lactose from the fabricated tablets was necessary, because some of its methylene protons overlapped with those of the −CH_2_– fragment of MED.

All systems exhibit the well-known “burst release” phenomenon, according to which an initial large bolus of drug is released before the release rate reaches a stable profile.^44^ The Na_2_–CLOD “reference system” exhibits an initial rate of 1.67 μmol/min, the Mg-CLOD-CP shows a slightly reduced rate at 1.32 μmol/min (by 19%), whereas the Sr–CLOD shows a substantially reduced initial rate (by 42%), 0.97 μmol/min. The slowest release is noted for the Ca-CLOD system, 0.70 μmol/min (by 58%). Furthermore, only 8.5 h are needed for the Na_2_–CLOD system to reach equilibrium (plateau) of 83%, while the t_p_ is 29.9 h for the Sr–CLOD and 25 h for the Mg-CLOD-CP to reach the plateau values of 90% and 100%, respectively. Finally, the t_p_ is ∼ 144 h for the Ca–CLOD, reaching the plateau of 94%.

All metal-containing release systems exhibit lower initial rates compared to Na_2_–CLOD. We assign the slower release kinetics of CLOD from the metal–CLOD systems to the hydrolysis of the metal-O (phosphonate) bonds, a requirement for the detachment of CLOD molecules from the coordination network and their subsequent dissolution into the aqueous phase. All factors affecting the release of CLOD will be discussed below in detail.

MED coordination with Ca^2+^ also slows retards the initial rate of release by ∼2.5 times (Table 3) and reaching the release plateau requires longer time, roughly by a factor of ∼7. Interestingly, the plateau value for Ca-MED (91%) is higher than the value for MED acid (72%). Factors affecting the release of MED will be discussed below.

### Drug Release Kinetics of the CLOD-Containing Systems

The study of the release kinetics was conducted using DDSolver software, a Microsoft Excel plug-in that provides access to 40 different kinetic models.^45^ The coefficient of determination (r^2^), Akaike Information Criterion (AIC) and Model Selection Criterion (MSC) criteria were employed to evaluate a model’s goodness of fit. The kinetic models used for fitting the release data included zero-order, first-order, Higuchi, Korsmeyer-Peppas, Hixson-Crowell, Peppas-Sahlin, Hopfenberg, Baker Lonsdale, and Weibull models. The results of this analysis are presented in Table 4.

**Table 4:** Release Kinetics Evaluation for the Na_2_–CLOD, Mg-CLOD-CP, Ca-CLOD, Ca-CLOD-CP, and Sr-CLOD Tablets and Calculated Parameters for Each Kinetic Model^a^

**Kinetics models**	**Criteria**	**Na_2_–CLOD**	**Sr-CLOD**	**Mg-CLOD-CP**	**Ca-CLOD**	**Ca-CLOD-CP**
**Zero-order** *F* = *k*_0_ · t	*k*_0_	2.82	1.46	1.157	0.374	0.678
	r^2^	–2.580	–0.521	–1.2359	0.1832	–1.3298
	AIC	125.16	139.83	159.3800	189.0146	165.1719
	MSC	–2.27	–0.87	–1.2973	–0.1546	–1.3342
**First-order** *F* = 100 · [1-Exp(-*k*_1_ · t)]	*k*_1_	0.33	0.12	0.180	0.028	0.104
	r^2^	0.858	0.956	0.9912	0.9667	0.8032
	AIC	86.46	90.35	76.3017	128.2293	125.6289
	MSC	0.95	2.66	4.2412	3.0446	1.1372
**Higuchi** *F* = k_H_ · t^0.5^	k_H_	18.85	13.14	12.936	6.523	9.159
	r^2^	–0.302	0.629	0.2699	0.8138	0.1965
	AIC	113.02	120.07	142.5925	160.9192	148.1936
	MSC	–1.26	0.54	–0.1781	1.3241	–0.2697
**Korsmeyer-Peppas** *F* = k_KP_ · t^n^	k_KP_	55.56	30.58	41.371	16.278	34.415
	r^2^	0.822	0.922	0.8845	0.9328	0.9393
	AIC	91.12	100.19	118.9298	143.5560	108.8049
	MSC	0.56	1.96	1.3994	2.2379	2.1887
**Hixson-Crowell** *F* = 100 · [1-(1-k_HC_ · t)^3^]	k_HC_	0.03	0.02	0.012	0.004	0.008
	r^2^	–0.222	0.944	0.0791	0.8210	0.0893
	AIC	112.26	119.97	146.0749	160.1692	150.1419
	MSC	–1.20	0.55	–0.4103	1.3636	–0.3949
**Peppas-Sahlin** *F* = k_PS(1)_ · t^m^ + k_PS(2)_ · t ^(2 · m)^	k_PS(1)_	50.99	23.22	33.878	10.107	28.518
	*k*_ps(2)_	–7.05	–1.38	–2.628	–0.259	–2.188
	r^2^	**0.946**	**0.990**	0.9710	**0.9885**	**0.9923**
	AIC	78.71	73.48	98.2278	112.0465	77.7546
	MSC	1.60	3.87	2.7795	3.8963	4.1293
**Hopfenberg** *F* = 100 · [1- (1 - k_HB_· t)^n^]	k_HB_	0	0	0	0	0
	r^2^	0.858	0.956	**0.9961**	0.9667	0.8032
	AIC	88.46	92.36	78.3149	130.2367	127.6316
	MSC	0.78	2.52	4.1070	2.9390	1.0120
**Baker-Lonsdale** $\frac{3}{2}$ [1-(1 - $\frac{F}{100}$ )^2/3^ ] - $\frac{F}{100}$ = k_BL_ · t	k_BL_	0.01	0.005	0.003	0.001	0.003
	r^2^	0.339	0.881	0.3967	0.9399	0.6305
	AIC	104.89	104.17	139.7295	139.4339	135.7091
	MSC	–0.58	1.67	0.0127	2.4549	0.5072
**Weibull** *F* = 100 · {1-Exp[-((t-Ti)^β)/α^]}	r^2^	**0.938**	**0.989**	**0.9956**	**0.9980**	**0.9863**
	AIC	80.43	74.97	69.8862	78.5793	89.9860
	MSC	1.45	3.76	4.6689	5.6578	3.5524

a In all models, **F**: is the percentage (%) of drug released at time t, **k**_**0**_ **:** zero-order release constant, **k**_**1**_ **:** first-order release constant, **k**_**H**_ **:** Higuchi release constant, **k**_**KP**_ **:** release constant incorporating structural and geometric characteristics of the drug-dosage form, **n:** diffusional exponent indicating the drug-release mechanism, **k**_**HC**_ **:** Hixson-Crowell release constant, **k**_**PS(1)**_ **:** Peppas-Sahlin release constant (related to the Fickian kinetics), **k**_**PS(2)**_ **:** is the constant related to Case-II relaxation kinetics, **m:** is the diffusional exponent for a device of any geometric shape which inhibits controlled release, **k**_**HB**_ **:** Hopfenberg release constant, n: is 1, 2, and 3 for a slab, cylinder, and sphere, respectively, **k**_**BL**_ **:** Baker Lonsdale release constant, **α:** is the scale parameter which defines the time scale of the process, **β:** is the shape parameter which characterizes the curve as either exponential (β = 1; case 1), sigmoid, S shaped, with upward curvature followed by a turning point (β > 1; case 2), or parabolic, with a higher initial slope and after that consistent with the exponential (β < 1; case 3), **Ti:** is the location parameter which shows the lag time before the onset of the dissolution or release process and in most cases will be near zero, **AIC:** Akaike Information Criterion, **r** ^**2**^ **:** determination coefficient, **MSC:** Model Selection Criteria. Values shown in bold are better selections according to evaluation criteria.

The most suitable kinetic model for describing the release data was determined based on the highest r^2^ and MSC values, and the lowest AIC value. According to these criteria, Figure 11 illustrates the release curves of two kinetic models per system that better describe the release of CLOD from the tablets. The Peppas-Sahlin model emerged as the most appropriate kinetic model for the systems Na_2_–CLOD, Ca-CLOD, Ca-CLOD-CP, and Sr-CLOD. This model incorporates Fickian diffusion and relaxation (Case II) as two mechanisms to describe the release of drugs from polymeric devices.^46^ The Hopfenberg model was better for Mg-CLOD-CP.

**Figure 11:**
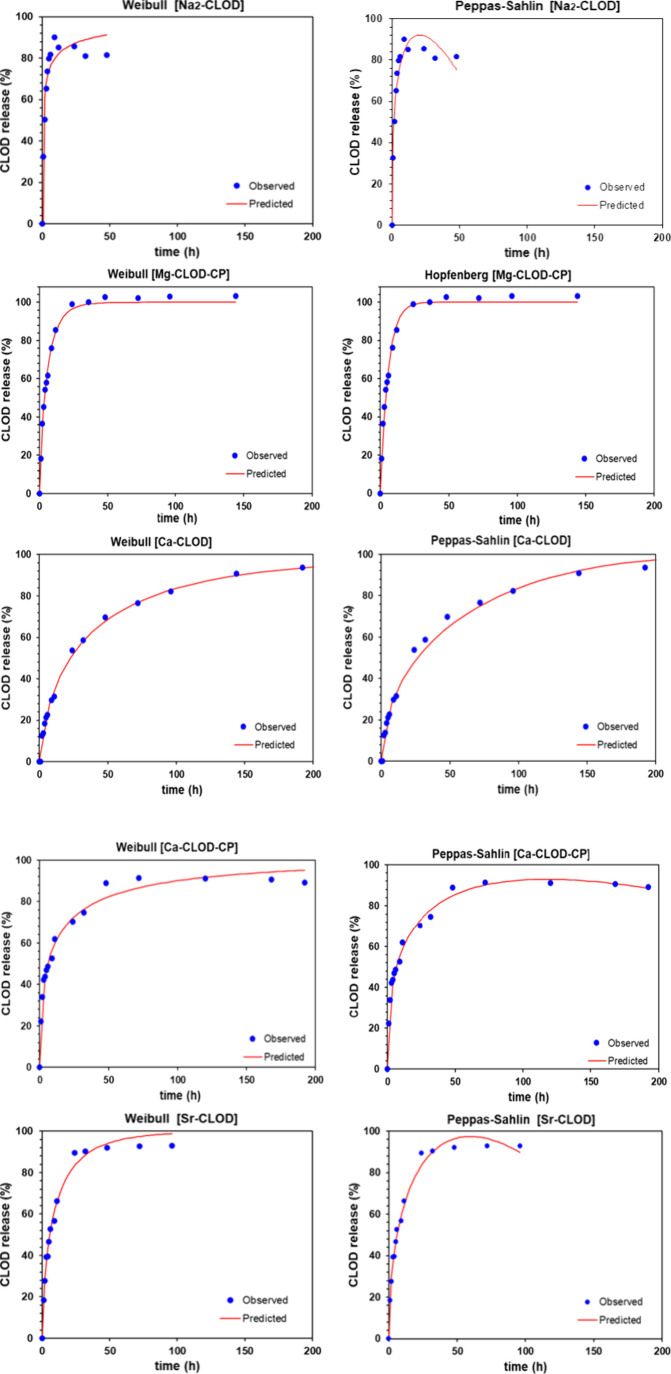
Observed data of CLOD release from the Na_2_–CLOD, Mg-CLOD-CP, Ca-CLOD, Ca-CLOD-CP, and Sr-CLOD tablets, along with the corresponding predictions from kinetic models. These kinetic models exhibit superior statistical parameters (r^2^, AIC, MSC) compared to the other models.

It is difficult to unequivocally classify the studied systems according to the well-established categories. This is because several events occur simultaneously or consecutively from the time of tablet exposure to the liquid phase until the active drug is finally released into the medium. At a first glance, they can be classified as “matrix” systems that are dissolution and diffusion controlled. According to this classification, the “active drug” (in our case the CLOD or MED “metallodrug”) is dispersed in a polymeric matrix. However, the latter (composed of three excipients) is not completely water insoluble, because lactic acid does partially dissolve (under these experimental conditions). Hence, in the present systems the rate-limiting step is controlled by both dissolution and diffusion. Because all three tablet components are hydrophilic, our systems could also be called swelling-controlled drug delivery systems, a subclass of water penetration-controlled drug delivery systems. Finally, one could argue that the systems that include Mg^2+^, Ca^2+^ or Sr^2+^ ions may be also classified as chemically controlled drug delivery systems, since a hydrolysis step of the Mg/Ca/Sr–O coordination bonds must precede the final drug release.

### Simultaneous Release of Metal Ions

Drug (CLOD or MED) dissolution/release from the tablets containing the metallodrug poses the question about the fate of the metal ions, i.e., whether they are also released into the supernatant fluid or remain incorporated into the tablet mass. To address this issue, we selected the Ca-CLOD system for which the release of Ca^2+^ ions was monitored over the course of 400 h. The results are shown in Figure S11 in the SI. Ca^2+^ ion release is similar to CLOD release for the first ∼ 10 h, but with a slightly slower initial rate, 0.51 μmol/min (compare that with 0.70 μmol/min for CLOD). The plateau value 52% is reached after ∼ 42 h (compare that with 94% after ∼ 144 h for CLOD). These results indicate that only 6% of CLOD and 48% of Ca^2+^ remain in the tablet after release is ceased and CLOD is preferentially dissolved over Ca^2+^.

### Tablet Characterization (Before and After Release)

As noted in the previous section, during the release experiments both CLOD and Ca^2+^ ions are released from the Ca-CLOD system and the results presented so far were obtained from solution measurements. Supporting Information can also be obtained from the characterization of the tablet, before and after release. Figure S12 in the SI presents SEM images of the tablet surface before and after the release experiment (400 h). The SEM images of the as-prepared tablet surface reveal a smooth surface and well dispersed Ca-CLOD particles (roughly 50–100 μm in size). The tablet surface after the release has the same appearance, but the Ca-CLOD particles have been substantially diminished in size due to dissolution.

The tablet bulk (before and after release) was characterized by powder X-ray diffraction. Figure S13 in the SI presents comparative XRD powder patterns of pure Ca-CLOD, a Ca-CLOD containing tablet before and after the release experiment (400 h). The diffraction peaks of pure Ca-CLOD are clearly visible in the XRD pattern of the tablet before release, as expected. After the release experiment all these peaks disappear and only broad signals around 2θ 15°, 23° and 35° remain (which were also present in the XRD pattern of the as-prepared tablet), likely due to the presence of the three excipients. It appears that the Ca-CLOD remaining in the tablet has been amorphized.

Lastly, Figure S14 in the SI presents elemental mapping and EDS spectra of the surface of a Ca-CLOD containing tablet before and after the release experiment (400 h). The Si:Ca and Si:P atom ratios before and after were compared (Si from the silica excipient is selected as a “reference” element, as silica has virtually no solubility at low pH values). The Si:Ca ratio was found ∼ 4:1 in the as-prepared tablet and ∼ 10:1 after release, confirming Ca^2+^ dissolution. Similarly, the Si:P ratio was found ∼ 2:1 in the as-prepared tablet and ∼ 5:1 after release, confirming CLOD dissolution. Interestingly, no Ca or P was detected in the interior of the tablet (a tablet broken in half was used for the study). This indicates that Ca^2+^ ions and CLOD have migrated and localized toward the tablet surface and below it.

### Toxicity Studies

Potential toxicity effects of Na_2_–CLOD, Mg-CLOD-CP, Ca-CLOD, Ca-CLOD-CP, and Sr-CLOD were evaluated on normal human fetal lung fibroblast (MRC-5) cells using the sulforhodamine B (SRB) assay after 48 h of incubation. Due to the low water solubility of the compounds under investigation, dimethyl sulfoxide (DMSO) was used as a solvent. It is important to note that DMSO exhibits cytotoxic effects at concentrations exceeding 1.5% v/v. To avoid solvent-related toxicity, the concentration of the tested compounds was limited to a maximum of 100 μM, corresponding to a final DMSO content of no more than 1% v/v, at which DMSO alone resulted in a cell viability of 78.2 ± 3.1%. At this concentration (100 μΜ), all tested compounds exhibited cell viabilities higher than 70%, specifically 76.6 ± 1.2% for Na_2_–CLOD, 85.4 ± 5.9% for Mg-CLOD-CP, 86.9 ± 3.9% for Ca-CLOD, 94.8 ± 5.7% for Ca-CLOD-CP, and 77.7 ± 3.3% for Sr-CLOD. According to ISO 10993–5, a compound is considered noncytotoxic if cell viability exceeds 70%, and therefore the observed DMSO concentration does not compromise the validity of the cytotoxicity evaluation.^25,26^ Accordingly, the tested compounds can be considered as substances of low or no toxicity at the concentration of 100 μM.

#### In Vitro Micronucleus Assay

The micronucleus (MN) assay is commonly employed to assess the genotoxic potential of compounds in normal cell lines. The formation of micronuclei serves as a biomarker for DNA damage and reflects the effects of mutagenic, genotoxic, or teratogenic agents,^25–28^ making it a reliable end point for quantifying chromosomal instability. Moreover, the MN assay shows a strong correlation with *in vivo* toxicological findings from animal studies [1–4], further supporting its relevance as a predictive tool in genotoxicity testing. In untreated MRC-5 cells, the percentage of micronuclei (MN) was 0.61 ± 0.10% (Figure 12). Following treatment with Na-CLOD, Mg-CLOD-CP, Ca-CLOD, Ca-CLOD-CP, and Sr-CLOD at a concentration of 100 μM, the frequency of micronuclei (MN) ranged from 0.86% to 1.22%, specifically: 0.86 ± 0.15% for Na-CLOD, 0.99 ± 0.12% for Mg-CLOD-CP, 1.06 ± 0.02% for Ca-CLOD, 0.94 ± 0.14% for Ca-CLOD-CP, and 1.22 ± 0.12% for Sr-CLOD. A slight increase in MN formation was observed in all treated groups compared with the control; however, in the case of Sr-CLOD, the MN frequency was approximately twice that of untreated cells, suggesting a modest but noticeable genotoxic effect at this concentration. The percentage of micronuclei observed in cell cultures after treatment with these agents appears to increase with their molecular weight (506.18 g/mol for Sr-CLOD, 373.03 g/mol for Ca-CLOD, 447.12 g/mol for Ca-CLOD-CP, 541.68 g/mol for Mg-CLOD-CP, and 360.92 g/mol for Na_2_–CLOD, respectively). Moreover, Sr-CLOD exhibits slightly higher lipophilicity and membrane permeability, attributable to the lower hydration energy of Sr^2^^+^, which promotes greater intracellular accumulation of the complex. Consequently, Sr-CLOD, which possesses both high molecular weight and strong cytotoxicity among the tested compounds (see Toxicity Studies above), also displays a higher proportion of small nuclear fragments compared with the other analogues.

**Figure 12:**
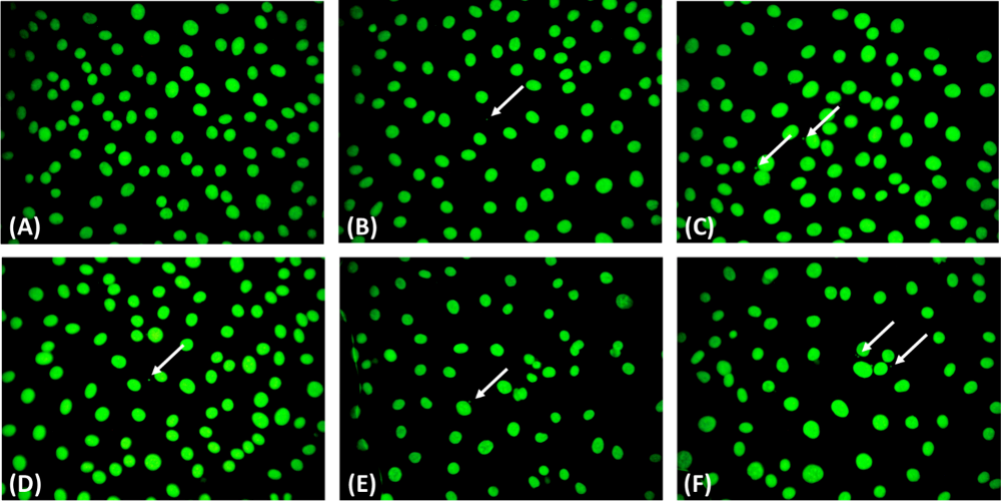
Representative images with micronuclei formed in nontreated MRC-5 cells (A) and treated with Na_2_–CLOD (Β), Mg-CLOD-CP (C), Ca-CLOD (D), Ca-CLOD-CP (E), and Sr-CLOD (F) (all at 100 μΜ concentration), for a period of 48 h. The white arrows indicate micronuclei in MRC-5 cells.

#### In Vivo Toxicity Evaluation by Brine Shrimp Artemia salina

*Artemia salina* is widely employed as an *in vivo* model organism for toxicological screening, in accordance with the United States Environmental Protection Agency (US-EPA) guidelines, as its toxicity responses have been shown to correlate well with those observed in rodent and human models.^26,29^ In this study, the *in vivo* toxicity of the CLOD compounds was evaluated at concentrations of 100, 200, and 400 μM. The survival rates of *Artemia salina* exposed to Na_2_–CLOD, Mg-CLOD-CP, Ca-CLOD, Ca-CLOD-CP, and Sr-CLOD at all tested concentrations remained at 100%. Therefore, all tested compounds can be considered nontoxic in this model, up to a concentration of 400 μM. At a compound concentration of 400 μM, the corresponding DMSO content in the complex solutions was 4% v/v. Notably, this level of DMSO did not exhibit any detectable toxicity toward *Artemia salina* larvae.

## Discussion

The use of metal-BP hybrid materials as BP controlled release systems requires knowledge of the precise structural identity of the drug-releasing compound. This allows the correlation of structure with function. An additional requirement is the purity of the compound to be used as a controlled release system, as impurities may lead to erroneous conclusions.

A successful synthesis outcome involves extensive experimentation with variables such as solution pH, reactant concentrations and molar ratios, temperature (and pressure, in the case of hydrothermal synthesis), etc. For the present research the bulk synthesis of pure, monophasic, and crystalline metal-CLOD products required extensive experimentation until the optimum synthesis pH was found for each system. Although the structures of Mg-CLOD-CP, Ca-CLOD, and Sr-CLOD have been published before, their synthesis procedure involved the use of silicate gels to grow a small amount of single crystals required for X-ray crystallography. This approach was unsuitable for the scope of the present research, mainly for two reasons: (a) it yielded insufficient quantities, and (b) the gel could not be effectively removed from the solid products. Hence, bulk syntheses were necessary, that were successful in giving satisfactory quantities of pure products.

The published crystal structures containing CLOD include its metal-containing forms. The crystal structure of clodronic acid is not known. The structure of the disodium salt tetrahydrate (Na_2_–CLOD used herein for the release studies) has been published.^47^ A mononuclear strontium complex of CLOD, Sr­[Cl_2_C­(PO_3_H)_2_(H_2_O)_5_], was published and it is the isostructural analog of Ca-CLOD.^48^ A Na/Mg derivative of CLOD was published, [NaMg­(Cl_2_CP_2_O_6_H)­(H_2_O)_5_]_n_, which is a coordination polymer.^21^ The mixed Na/Zn and Na/Cd derivatives of CLOD are also coordination polymers.^49^ Besides Na_2_–CLOD (studied herein) none of the above materials have been studied for CLOD release, because they were synthesized/crystallized in gels and their bulk synthesis was unsuccessful.

CLOD release experiments from fabricated tablet systems were carried out at pH 1.3. This value was selected to mimic the pH of the human stomach. We are aware of the fact that stomach fluid is a much more complicated system, which cannot be fully simulated in the laboratory. However, because metal phosphonate compounds are unstable at such low pH, it was decided that pH is the principal factor for the acid-driven hydrolysis of the M-O­(phosphonate) bonds and this approach is sufficient for a “proof-of-concept”.^31^ P­{^1^H} NMR spectroscopy was used for the quantification of CLOD in the supernatant phase, as there are no protons in the CLOD molecule that can be detected (and quantified) by ^1^H NMR. The latter technique was used successfully for the quantification of a plethora of BPs in our laboratory.^7–9,24,50–52^

The controlled release curves of the five CLOD-containing systems are shown in Figure 9 and kinetic data are compiled in Table 3. The results obtained show that all M^2+^-containing (M = Mg, Ca, Sr) CLOD systems exhibit a substantially lower initial release rates than the Na-containing “control” CLOD system, which is considered in the release studies to be the “free” drug system, used as the baseline (Na^+^ salts of BPs are generally very soluble). Using the data from Table 3, it is difficult to draw general trends. For example, an attempt could be made to correlate the initial rate data with the structural data in Table 2, e.g., the number of interactions (H-bonds and M-O bonds) “locking” the CLOD molecule in the crystal lattice. The hypothesis here is that the greater the number of interactions is, the lower the initial rate is. Based on this hypothesis, Ca-CLOD-CP (16 total interactions) should be the fastest CLOD-releasing system, and Mg-CLOD-CP (25 total interactions) the slowest one. The reverse trend is actually observed. Other factors that may affect the initial rate include the strength of the M-O­(phosphonate) bonds that must be hydrolyzed by acid for removal of the CLOD molecule from the crystal lattice and into the solution. The expected strength of the M-O­(phosphonate) bonds should follow the order Mg–O > Ca–O > Sr–O, but this is not reflected on the initial rates. The above arguments do apply, however, to the Na_2_–CLOD system, used as a “baseline” for the CLOD release, with this system displaying the fastest release.

Another factor that could be examined is the packing of each structure. It is expected that the denser the packing is the higher the initial rate is expected to be. The criterion used is the closest metal···metal contacts was applied for the Mg, Ca, and Sr structures. The following closest contacts were considered: the interchain Mg···Mg contact (12.710 Å) for Mg-CLOD-CP, the Ca···Ca contact (6.619 Å) between individual complexes for Ca-CLOD, the Ca···Ca contact (5.716 Å) for Ca-CLOD-CP, and the interlayer Sr···Sr contact (7.009 Å) for Sr-CLOD. The t_p_ values (time in ours for the plateau value to be reached) also show some correlation with this structural feature. In short, it appears that the denser the packing of a certain release system is, the slower the initial rate is and the longer it takes the CLOD release to reach the equilibrium value. These trends are plotted in Figure 13.

**Figure 13:**
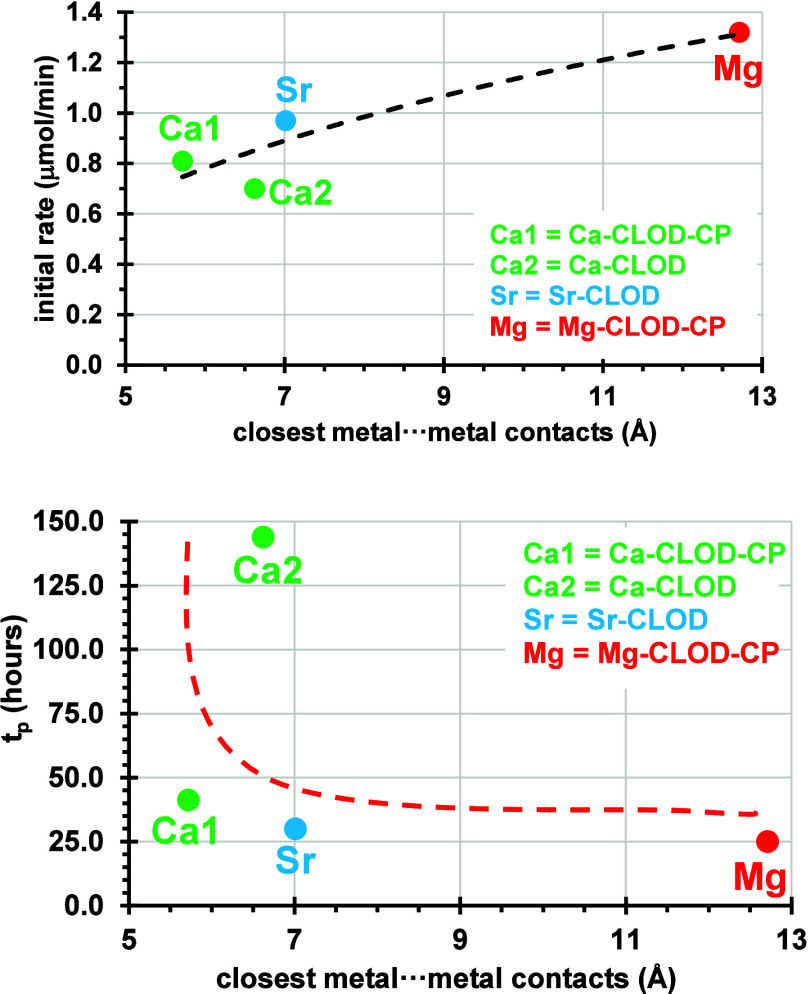
Correlations between initial rate of CLOD release values (upper) and the t_p_ times (lower) with the closest metal···metal contacts in the structures of Mg-CLOD-CP, Ca-CLOD, Ca-CLOD-CP, and Sr-CLOD. Lines are drawn to aid the reader. The same color coding for the compounds was used as in Scheme 1.

The slow release of the Ca-CLOD system is intriguing because, based on its structural features, it should be expected to display the fastest release. However, it shows a very low initial release rate of 0.70 μmol/min, with a plateau value of 94% reached in ∼ 144 h (Figure 7, Table 3). These results cannot be rationalized based on the compound’s structural features, shown in Table 2. There are only two coordination bonds between Ca and CLOD and 12 H-bonds per CLOD ligand. Hence, we propose that the governing factor that determines CLOD release, apart from the packing density highlighted above, is the shape and size of the crystallites of Ca-CLOD. It is reasonable to assume that these properties of the Ca-CLOD single crystals endow them with a slower release of the “active” CLOD.

Based on the SEM images shown in Figure 2, the Ca-CLOD single crystals are blocks that have a distinct polygonal shape, compared to the needle-shaped crystals of Mg-CLOD-CP or Sr-CLOD, with widths of ∼ 10 μm and 5–10 μm, respectively. Additional SEM images were recorded for the Ca-CLOD bulk material (see Figure S15 in the SI) and representative size measurements were done for 25 single crystals. These are depicted in Figure S16 in the SI. The minimum crystal size recorded was 76 μm, the maximum size was 477 μm, while the average size was 198 μm.

Τhe bulk drug-containing solid products undergo grinding before mixing with the excipients for tablet fabrication, with a final grinding step with all ingredients included, before that powder is pressed to a pill. Hence, SEM images of the ground solids were also recorded to gain more information on a rough particle size distribution. These images are shown in Figure S17 in the SI. In the Mg-CLOD-CP sample, most needle-shaped crystals have sizes below 5 μm. Similar observations can be made for the needle-shaped crystals of Sr-CLOD. However, the fragments of Ca-CLOD crystals have sizes >10 μm. All samples show a degree of size variability, and particles are not uniform. Although the above observations are qualitative, they are consistent with the fact that Ca-CLOD demonstrates lower initial dissolution rates than the other systems.

Although there are no sufficient data to conclusively assign the observed release data to particle shape and size, it becomes apparent that this factor must be considered in interpreting release data. For this, a suitable material is required, that is available in systematically variable particle sizes, each batch of which having a narrow particle size range (ideally uniform). Such efforts are underway in our laboratory in order to unequivocally confirm the effect of particle size on drug release rates.

It is reasonable to assume that the CLOD “active” drug is released into the aqueous medium in the fully protonated, acid form. The pH of the release experiments is 1.3, lower than its p*K*_a1_ value (1.7). Furthermore, the scenario that water-soluble metal-CLOD complexes are among the species released from the tablet is not likely. The reasons for this are (a) the very low pH medium, and (b) the single phosphorus signal in the ^31^P­{^1^H} NMR spectrum. Complexation of CLOD to metal ions in solution may cause peak shift, or broadening. Such observations were not noted in any of the working solutions.

Factors that may influence the release features of a specific drug release system include intrinsic and extrinsic ones. The former are (a) the solubility of the “active” drug itself, (b) the structure packing density, (c) the strength and number of the interactions holding the “active” drug in the structure (M-O and H-bonds), (d) the properties of the metal ions that are coordinated with the drug, (e) the presence and number of lattice water molecules. The latter include: (a) the pH of the release aqueous medium, (b) the temperature, (c) the presence/absence of certain excipients in the tablet, and their physicochemical properties (e.g., swelling), (d) the particle size of the drug release systems crystallites in the tablet. It seems like an impossible task to systematically study the effect of all these factors on drug release systems and draw reliable conclusions based on structure function relationships. Unavoidably, such an endeavor must be implemented fragmentally, and step-by-step. The most important challenge is the availability of precisely characterized candidate compounds for drug release that show systematic differences (e.g., metal ions with systematically increasing ionic radius), but keeping other structural features identical (e.g., structure dimensionality). All these challenges, however, should not discourage efforts to map the release features of such systems because the driving force is the actual application, i.e., the development and use of such systems for disease treatment.

The release data of MED and Ca-MED systems warrants some comments. These are 2-excipient systems containing only cellulose and silica (no lactose). Recently, we performed a systematic study on the various factors affecting release in “free” BP systems (no metal present).^24^ The presence of lactose particles in tablets improves the drug’s dissolution after being compressed.^53^ The proposed role of lactose in the tablet formulations is to assist in water (solvent) penetration into the tablet, hence inducing swelling, partial lactose dissolution and tablet erosion, thus exposing the BP particles that are dispersed in the bulk tablet. No chemical interactions between lactose (or the other excipients) with the drug are anticipated in the solid state (tablet). Our published data pointed to the fact that when lactose is not included in the tablet (2-excipient systems), initial rates, plateau values and t_p_ values are systematically lower (but variable, depending on the individual BP) compared to the 3-excipient systems. The presence of Ca^2+^ in Ca-MED undoubtedly retards dissolution of “free” MED, but the kinetic data presented in Table 4 are underestimated because of the absence of lactose. Hence, Ca-MED would be expected to show faster release, if it were possible to monitor a 3-excipient system. This is consistent with the structural data in Table 3, with MED forming a total of 12 interactions in the lattice. Notwithstanding the above, comparisons of BP release features between 3- and 2-excipient systems should be avoided for structurally different BPs.

The present work supplements BP controlled delivery from different systems. Some notable examples are warranted. Titanium implants coated with calcium zeolite were used as controlled delivery systems for the BP drug risedronate.^54^ It was found that one year is needed for the release of 30% of the total drug quantity. Biphasic calcium phosphate (BCP) scaffolds were loaded with the BP drug alendronate (ALE).^55^ The drug release notwithstanding, the osteogenetic activity in MG-63 cells and mineralization *in vivo* based on a rat tibial defect model were evaluated. The drug release was dose dependent, and the ALE/BCP scaffolds operated as enhancers for bone formation. Three isotypical coordination complexes with the general chemical formula {[M_2_(H_4_ALE)_4_(H_2_O)_2_]·1.5H_2_O} were fabricated by the combination of the drug ALE with various amounts of Mg^2+^, Ca^2+^, or Sr^2+^ cations. The therapeutic action of these compounds was found to be dependent on the long release period and also on the contribution of the released metal ions in order to improve the osteoblast metabolic activity.^56^ Direct and reliable comparisons between the metallodrug systems studied here with previously published ones should be avoided because other BP drugs (ETID,^7^ PAM,^7^ ALE,^7^ RIS,^8^ ZOL^9^) were studied with dramatic structural differences with CLOD or MED.^24^ In view of the low bioavailability of BPs in general (e.g., the bioavailability of CLOD is 1–2%),^57^ controlled release approaches will assist in effectively delivering the BP active drug, while avoiding “mega” doses that create undesirable side effects.^58^

## Conclusions

The main findings of the present study are as follows:

1. Convenient bulk syntheses of high purity M-CLOD (M = Mg, Ca, Sr) and M-MED (M = Ca) compounds were reported. All have been structurally characterized. The crystal structure of a new Mg dimeric complex, Mg_2_[(Cl)_2_C­(OH)­(PO_3_)_2_(H_2_O)_7_]·5H_2_O, which appears as a byproduct in the synthesis of Mg-CLOD-CP, was reported that exhibits a Mg-μ-H_2_O–Mg bridge with unusually long (2.425 Å) Mg–O bond distance.
2. The Na_2_–CLOD (used as a reference), Mg-CLOD-CP, Ca-CLOD, Ca-CLOD-CP, and Sr-CLOD compounds were included in controlled release systems (excipient-containing tablets) and the release of the active drug CLOD was studied under conditions that mimic the human stomach (pH = 1.3).
3. The drug release profiles of the four compounds were compared, and it was found that all Mg/Ca/Sr-containing systems exhibit a deceleration of the “active” CLOD, compared to the reference system Na_2_–CLOD. The order of increasing initial rate of CLOD release based on the cation was found to be Na^+^ > Mg^2+^ > Sr^2+^ > Ca^2+^.
4. Efforts were put forth to rationalize this behavior based on the structural idiosyncrasies of each system. The overall drug release profile for each system was the result of several structural factors, such as H-bonding interactions and strength of the metal–O­(phosphonate) bonds, density of packing, and particle size of the metal-CLOD crystallites.
5. Although some of the above factors were able to explain certain kinetic data, some results stand out. The structural analysis cannot explain the low initial release rates observed for the Ca-CLOD system. The only semiqualitative explanation is that the particle sizes of the crystalline fragments of Ca-CLOD present in the tablets are larger (>10 μm) than the other systems.
6. All CLOD-containing compounds reported here were tested for *in vitro* (micronucleus assay) and *in vivo* (brine shrimp *Artemia salina*) toxicity and were found to be of low toxicity.

Based on these results, it is concluded that factors such as the nature of the metal cation in such coordination compounds, the crystal packing, but also the size and shape of the metal-BP particles apparently influence both the initial drug release rates and the final plateau value.

## Supplementary Material











## Notes

ATR-IR spectra, powder XRD diagrams, TGA curves, ^1^H and ^31^P NMR spectra, SEM images (PDF)
Cif file for Mg-CLOD-D (CCDC no. 2340861) (CIF)
Cif file for Ca-CLOD-CP (CCDC no. 2369174) (CIF)
Cif file for for Ca-MED (CCDC no. 2410009) (CIF)
Datablock EC1—ellipsoid plot (PDF)
