# Supplementary material for: Antiosteolytic Bisphosphonate Metallodrug Coordination Networks: Dissolution Profiles and In Vitro/In Vivo Toxicity toward Controlled Release
Source: ACS Appl Bio Mater. 2025 Nov 18;8(12):11206–23. doi: 10.1021/acsabm.5c01890 (PMC12709581; doi:10.1021/acsabm.5c01890)
Supplement: Supplementary file 1 [file mt5c01890_si_001.pdf]

## Supporting Information

## For

# Anti-osteolytic bisphosphonate metallodrug coordination networks: Dissolution profiles and *in vitro/in vivo* toxicity towards controlled release

By

*Elpiniki Chachlaki,[a] Maria Vassaki,[a] Petri A. Turhanen,[b] Duane Choquesillo-Lazarte,[c]*

*Christina N. Banti,[d] Sotiris K. Hadjikakou,[d,e] and Konstantinos D. Demadis*[a]*

*Email: demadis@uoc.gr

[a] Crystal Engineering, Growth and Design Laboratory, Department of Chemistry, University of Crete, Voutes Campus, Heraklion, Crete, GR-71003, Greece

[b] University of Eastern Finland, School of Pharmacy, Biocenter Kuopio, P.O. Box 1627, FIN-70211, Kuopio, Finland

[c] Laboratorio de Estudios Cristalográficos, IACT, CSIC-Universidad de Granada, Granada-18100, Spain

[d] Laboratory of Biological Inorganic Chemistry, Department of Chemistry, University of Ioannina, Ioannina, GR-45110, Greece

[e] Institute of Materials Science and Computing, University Research Center of Ioannina (URCI), Ioannina, GR-45110, Greece

**Table S1.** Crystal data for the compounds Mg–CLOD-D, Ca-CLOD-CP, and Ca-MED.

| | Mg–CLOD | Ca-CLOD-CP | Ca-MED |
|---|---|---|---|
| **Empirical formula** | $CH_{26}Cl_2Mg_2O_{19}P_2$ | $CH_{14}Ca_2Cl_2O_{13}P_2$ | $CH_8CaO_8P_2$ |
| $M_r$ | 523.68 | 374.55 | 203.59 |
| **Crystal system** | monoclinic | monoclinic | monoclinic |
| **Space group** | C2/c | P21/n | P21/n |
| **a (Å)** | 13.5126(4) | 8.8023(6) | 9.906(2) |
| **b (Å)** | 16.5209(5) | 14.6502(12) | 6.9639(17) |
| **c(Å)** | 10.3783(5) | 11.8998(9) | 12.299(3) |
| **α (°)** | 90 | 90 | 90 |
| **β (°)** | 119.6670(10) | 106.469(3) | 98.621(8) |
| **γ (°)** | 90 | 90 | 90 |
| **V (Å$^3$)** | 2013.15 | 1471.59 | 838.853 |
| **Z** | 4 | 4 | 4 |
| **R factor (%)** | 3.29 | 4.32 | 5.44 |
| **CCDC code** | 2340861 | 2369174 | 2410009 |

## Quantification of $Ca^{2+}$ ions with the Titration Method

***Reagent solutions for the titration of $Ca^{2+}$ ions.*** The following solutions were prepared: (a) A 0.001 M EDTA solution was prepared by dissolving 0.190 g of $Na_4$EDTA in 500 mL deionized water. (b) A NaOH (2 M) solution was prepared by dissolving 20.000 g of NaOH in 250 mL deionized water water. (c) The Calcon indicator solution was prepared by dissolving 0.100 gr of Calconcarboxylic acid in 20 mL of methanol.

***Experimental Procedure.*** [a,b] A burette is filled with EDTA 0.001 M solution. Then a 350 μL sample of the working solution (the supernatant to which the tablet is exposed to), 5 mL distilled water, 50 μL of 2 M NaOH solution and 4 drops of the Calcon indicator are added in an Erlenmeyer flask, followed by slow addition of the EDTA from the burette, with continuous swirling until the indicator's color is turned completely from pink to blue. The total quantity (milliliters) of EDTA used is noted. The concentration of the $Ca^{2+}$ ions in the analyzed sample is calculated based on the volume of EDTA solution consumed until the color change.

[a] Eaton, A.D.; Clesceri, L.S.; Rice, E.W.; Greenberg, A.E.; Franson, M.H. *Standard Methods for Examination of Water & Wastewater*, American Public Health Association, Washington DC, **2005.** [b] Patton, J.; Reeder, W. New Indicator for Titration of Calcium with (Ethylenedinitrilo) Tetraacetate. *Anal. Chem.* **1956**, *28*, 1026-1028.

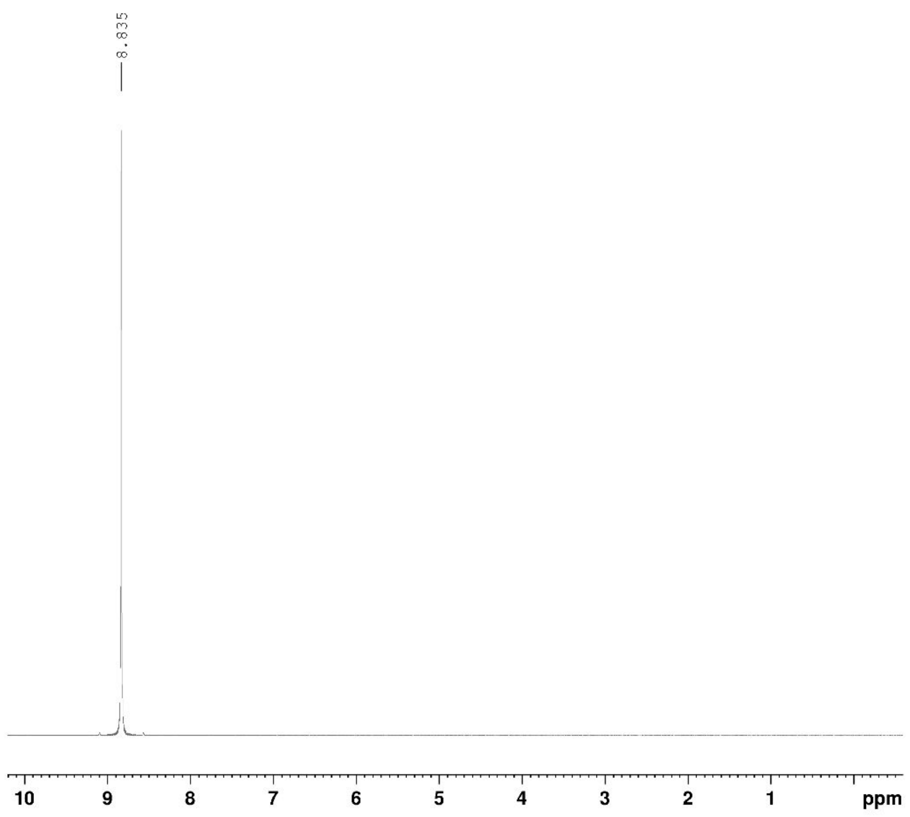


**Figure S1.** $^{31}P\{^1H\}$ NMR spectrum of CLOD disodium tetrahydrate in $D_2O$.

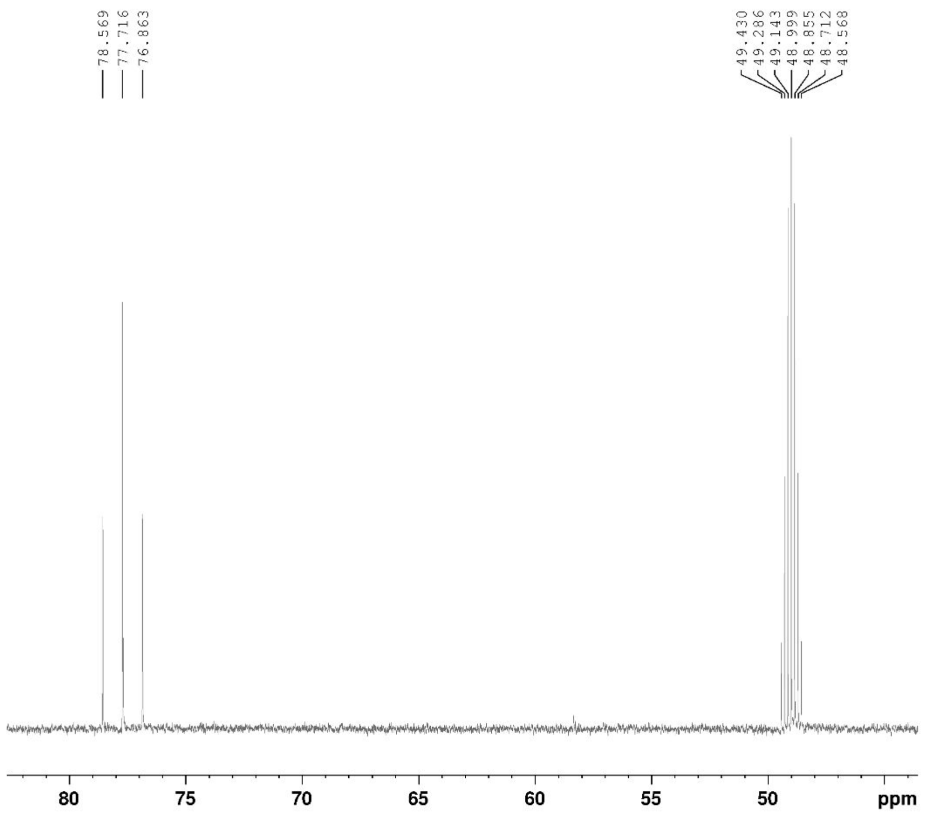


**Figure S2.** $^{13}C\{^1H\}$ NMR spectrum of CLOD disodium tetrahydrate in $CD_3OD$.

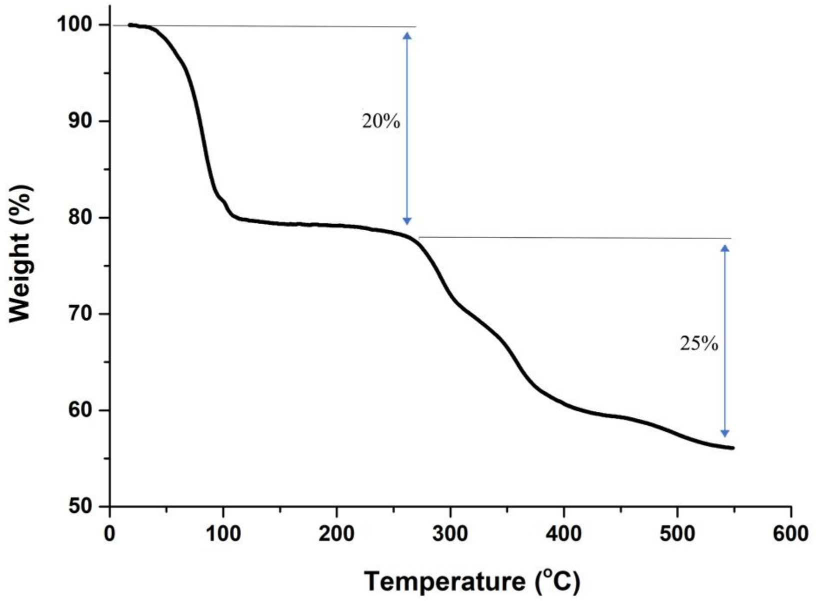


**Figure S3.** Thermogravimetric analysis of disodium clodronate tetrahydrate ($Na_2$-CLOD).

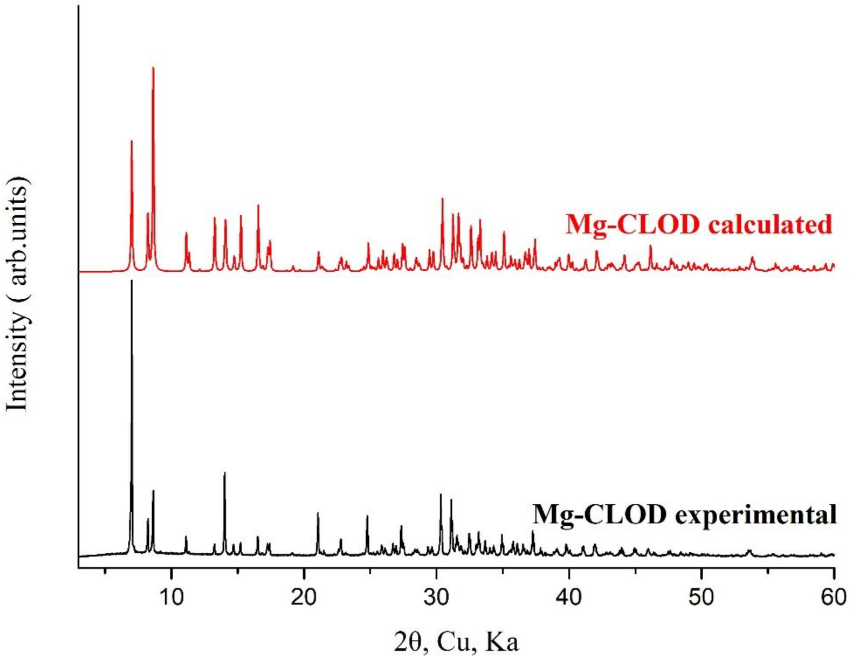


**Figure S4.** Experimental and calculated powder XRD petterns for the compound Mg-CLOD-CP.

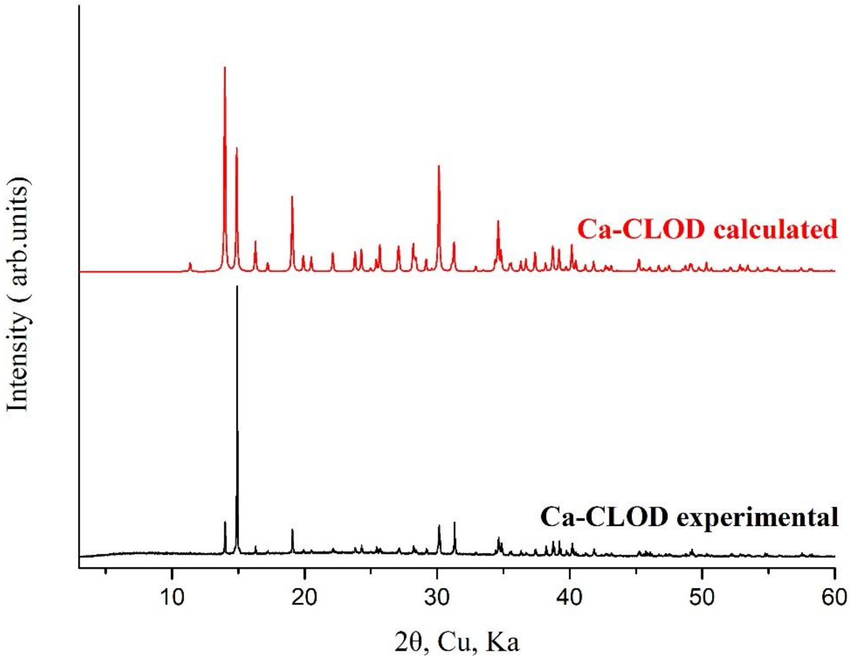


**Figure S5.** Experimental and calculated powder XRD petterns for the compound Ca-CLOD.

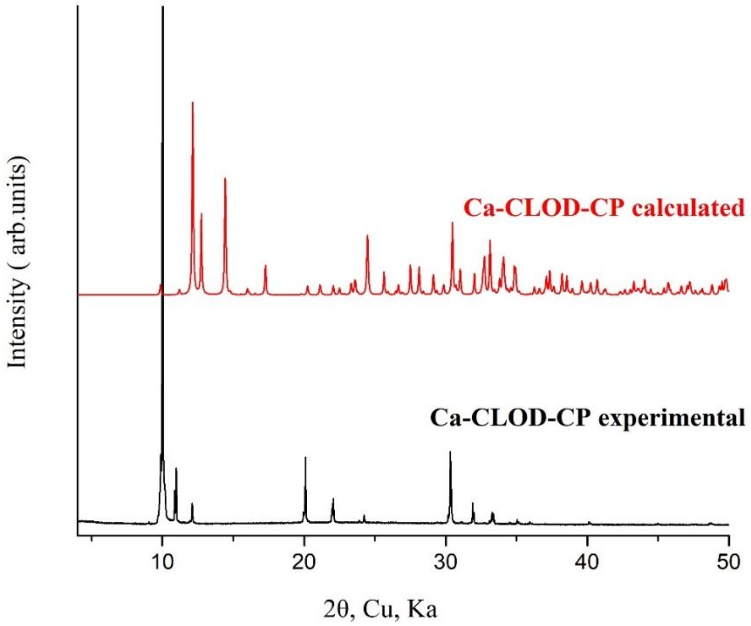


**Figure S6.** Experimental and calculated powder XRD petterns for the compound Ca-CLOD-CP.

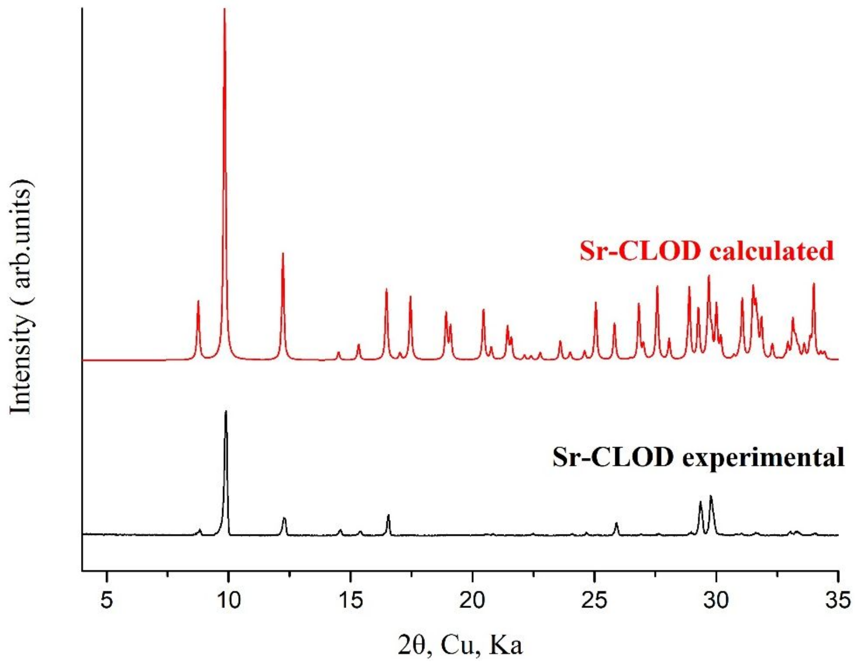


**Figure S7.** Experimental and calculated powder XRD petterns for the compound Sr-CLOD.

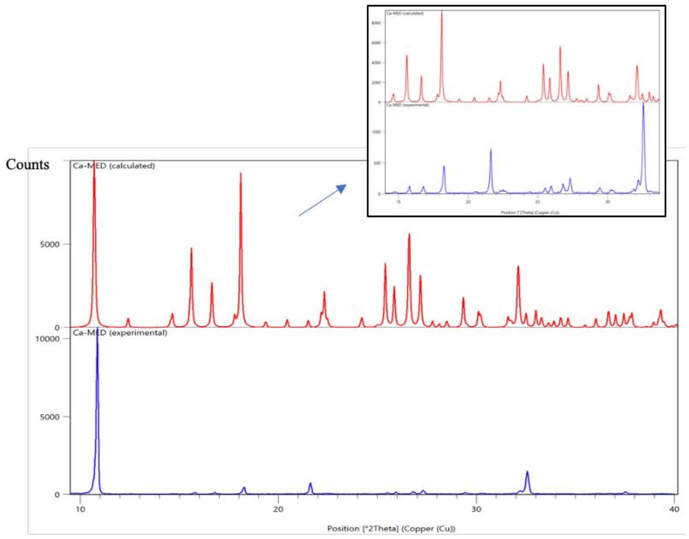


**Figure S8.** Experimental (blue) and calculated (red) powder XRD petterns for the compound Ca-MED.

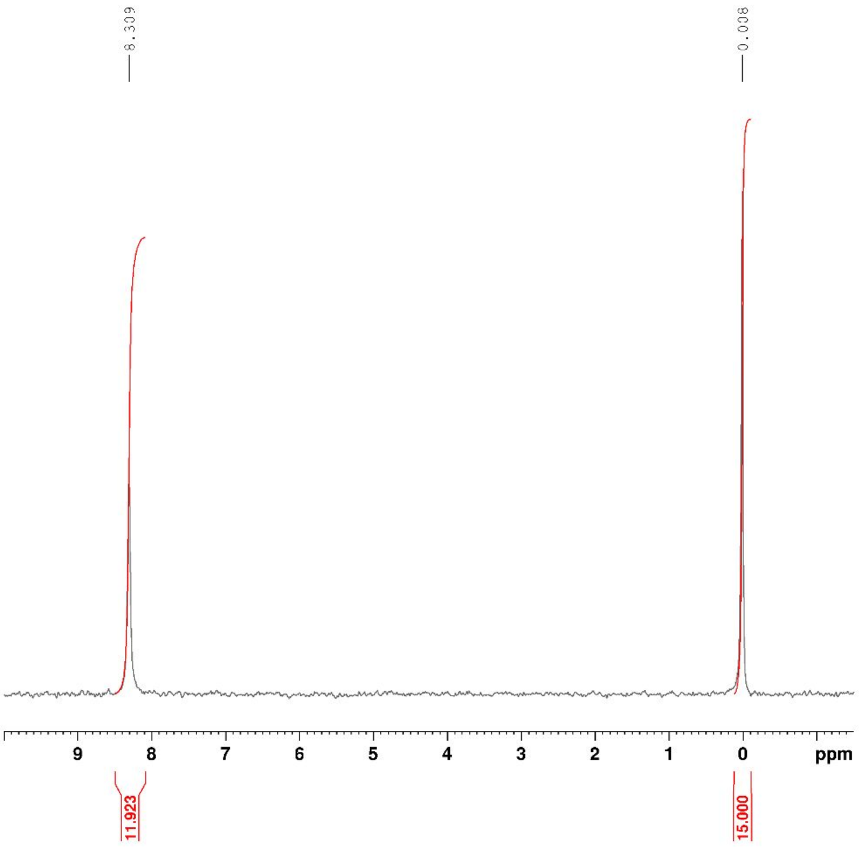


**Figure S9.** Representative $^{31}P\{^{1}H\}$ NMR spectrum of quantification of CLOD , integral of CLOD (δ 8.309) compared to the integral of standard solution of $KH_2PO_4$ (δ 0.008).

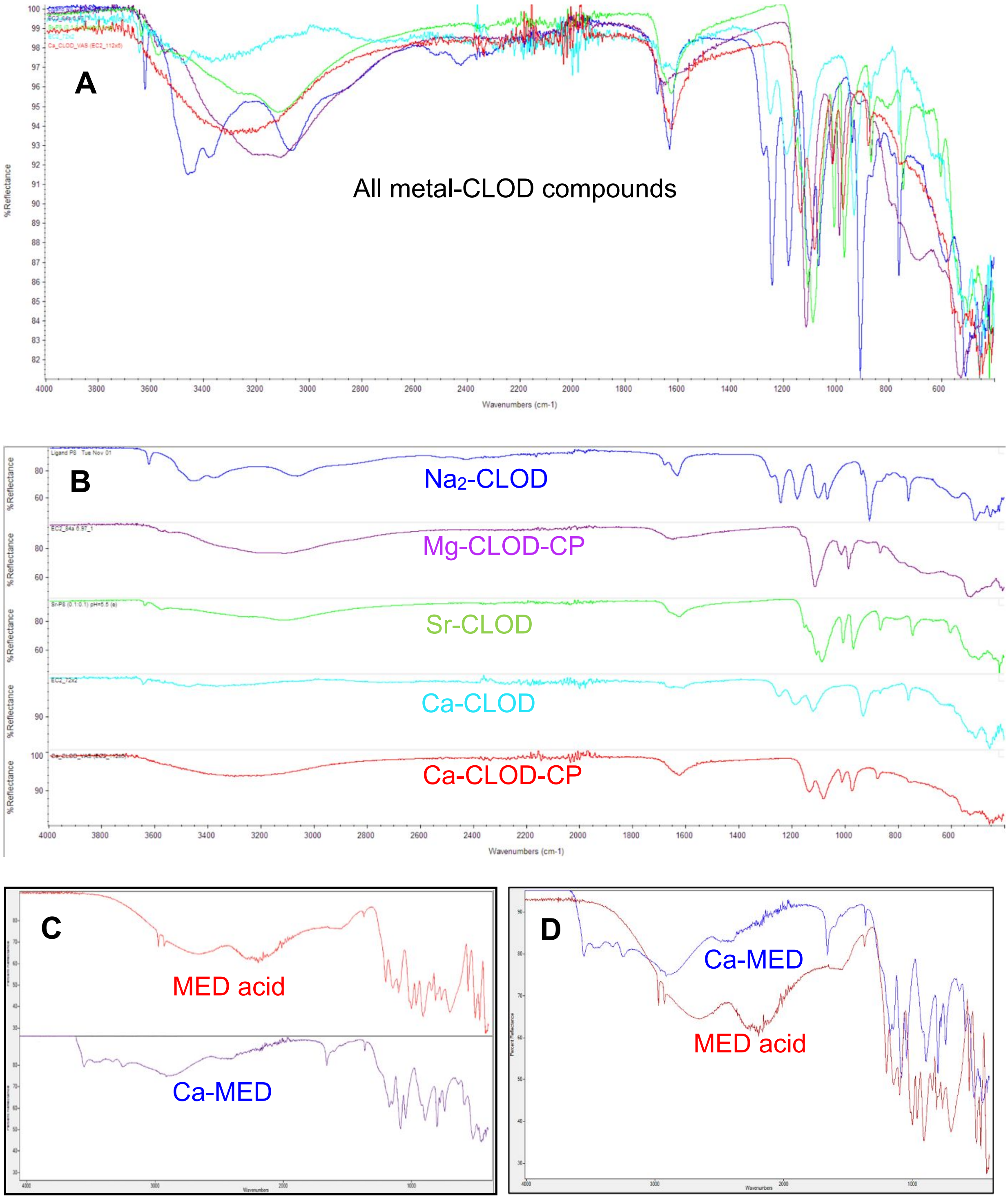


**Figure S10.** ATR-IR spectra. (A) Overlay spectra of metal-CLOD compounds. (B) Stack spectra of metal-CLOD compounds. (C) Stack spectra of MED acid and Ca-MED. (D) Overlay spectra of MED acid and Ca-MED.

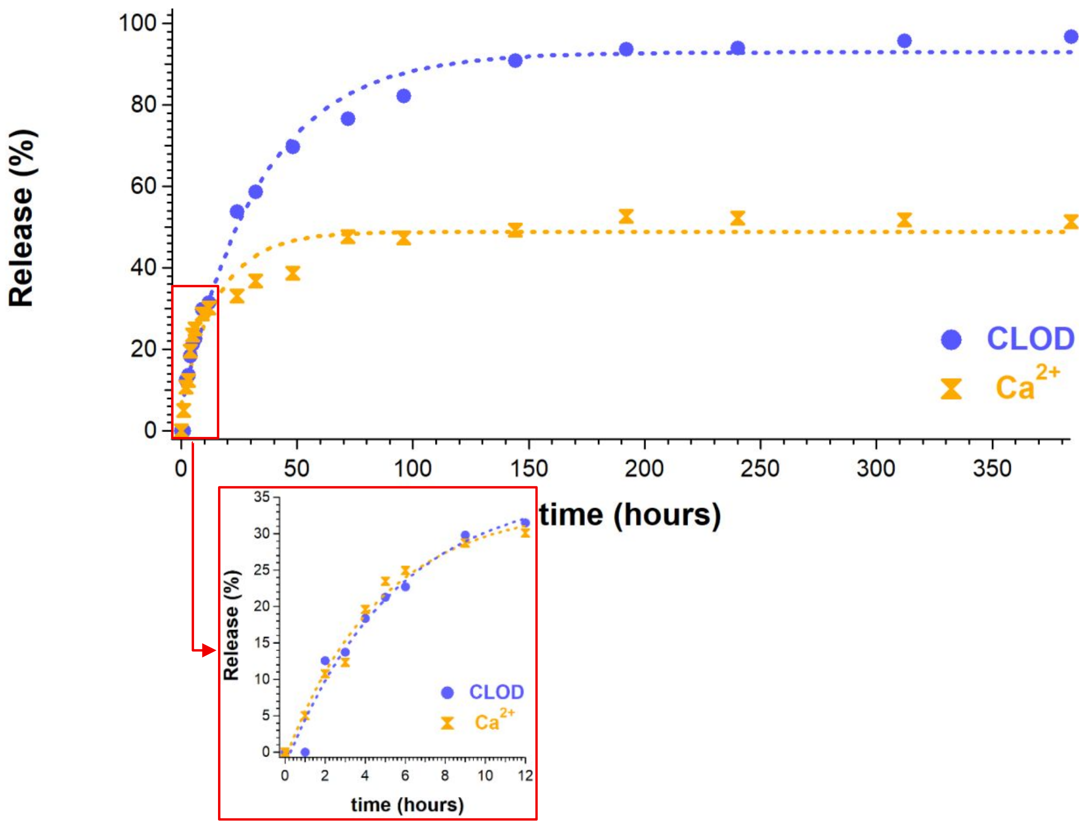


**Figure S11.** Comparison of the simultaneous release of CLOD and $Ca^{2+}$ ions from a Ca-CLOD containing tablet.

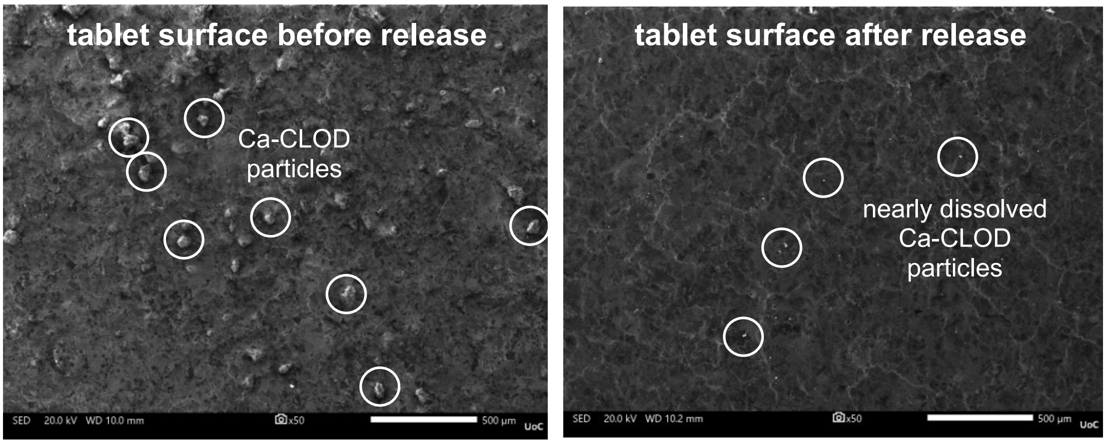


**Figure S12.** SEM images of a Ca-CLOD containing tablet surface before (left) and after (right) the release experiment. Representative Ca-CLOD particles are shown in circles.

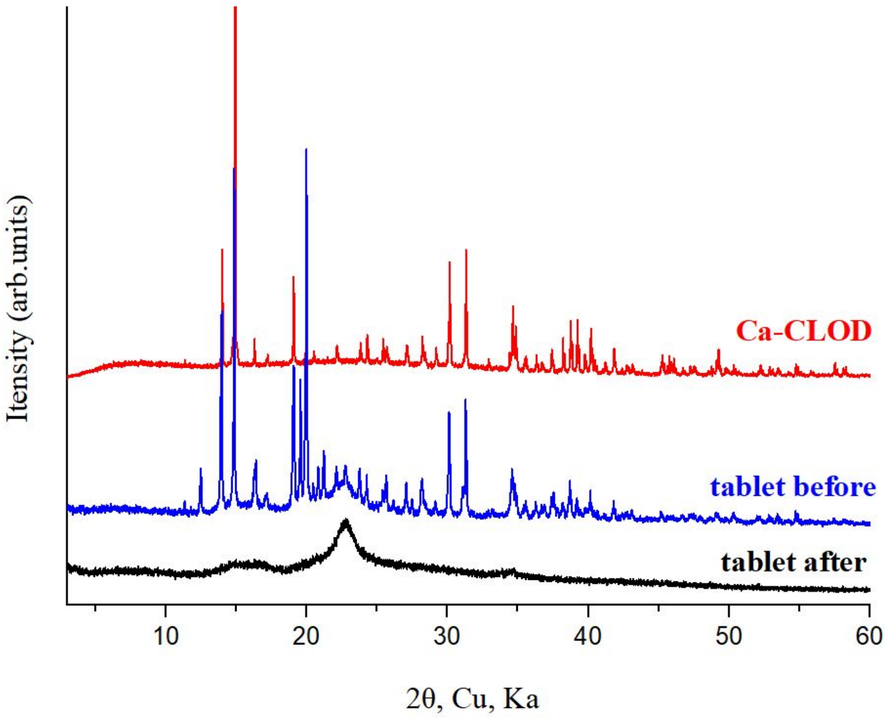


**Figure S13.** XRD powder patterns of pure Ca-CLOD (red), a Ca-CLOD containing tablet before (blue) and after (black) the release experiment.

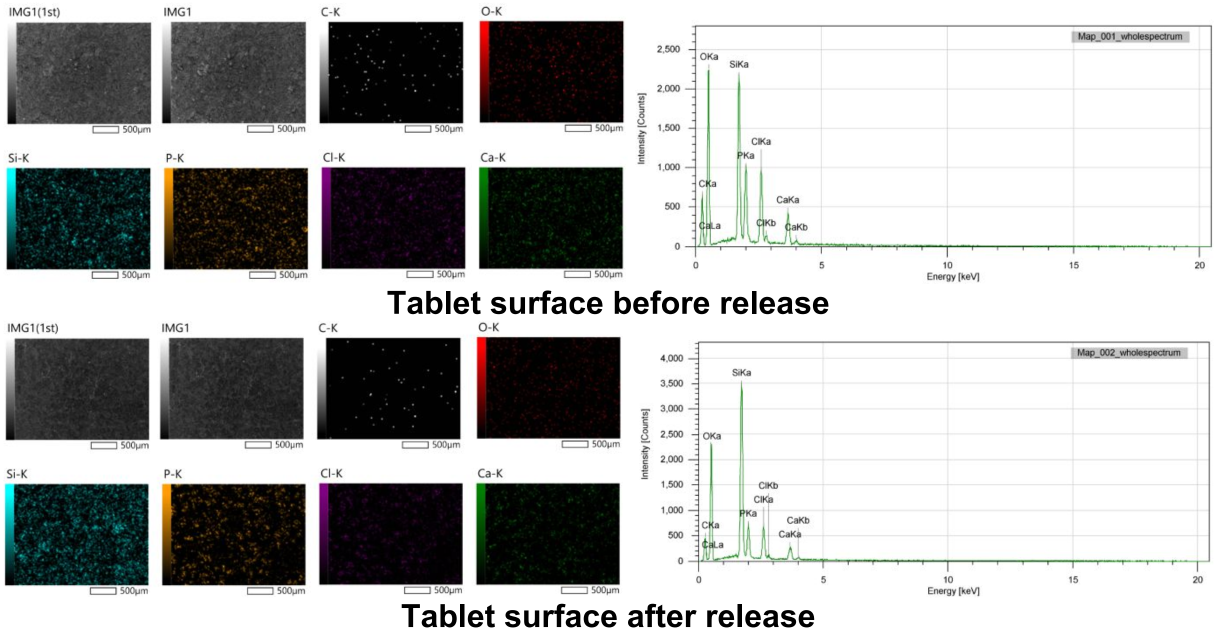


**Figure S14.** Elemental mapping (left) and EDS (right) results of a Ca-CLOD containing tablet before (upper) and after (lower) the release experiment (400 hours).

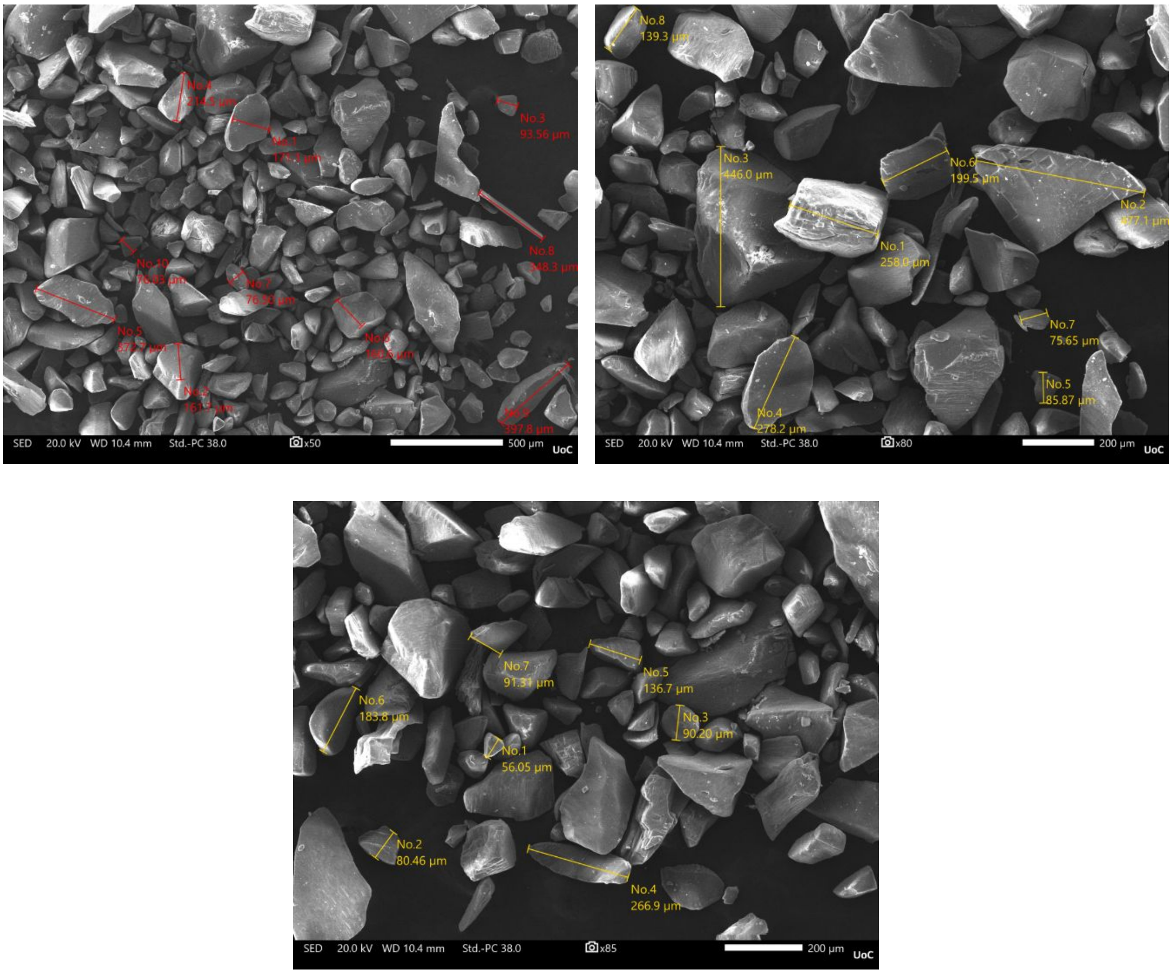


**Figure S15.** Representative SEM images of the Ca-CLOD bulk material, showing several size measurements.

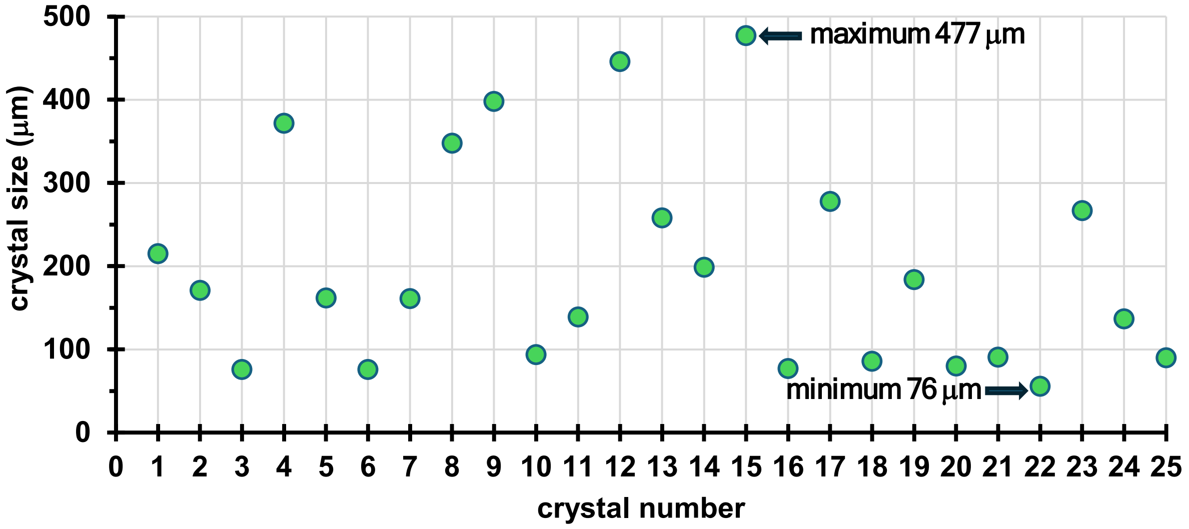


**Figure S16.** Size measurements of the Ca-CLOD bulk material, showing the minimum, the maximum and the average crystal size.

| Compound | Image 1 | Image 2 | Image 3 |
|---|---|---|---|
| **Mg-CLOD-CP** | 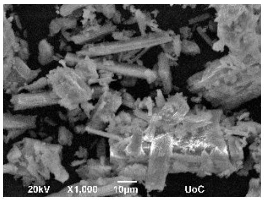 | 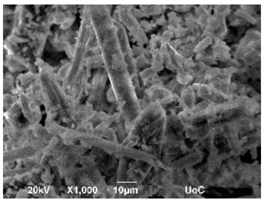 | 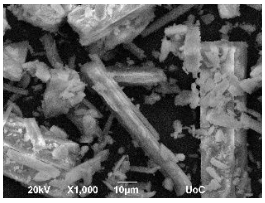 |
| **Ca-CLOD** | 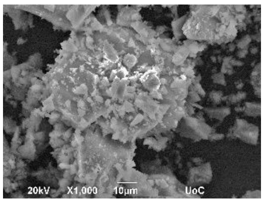 | 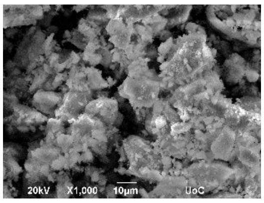 | 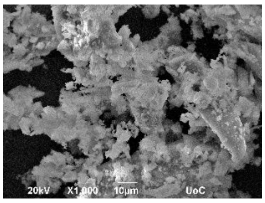 |
| **Sr-CLOD** | 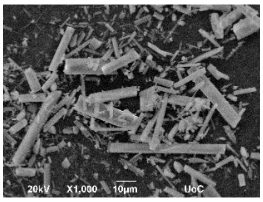 | 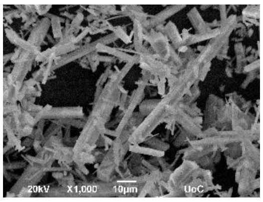 | 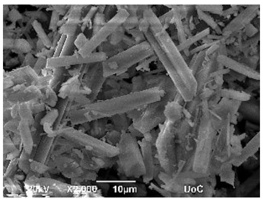 |

**Figure S17.** Representative SEM images of crushed (mortar-and-pestle) samples of Mg-CLOD-CP, Ca-CLOD, and Sr-CLOD that were used in the tablet fabrication.
