# Supplementary material for: Antiosteolytic Bisphosphonate Metallodrug Coordination Networks: Dissolution Profiles and In Vitro/In Vivo Toxicity toward Controlled Release
Source: ACS Appl Bio Mater. 2025 Nov 18;8(12):11206–23. doi: 10.1021/acsabm.5c01890 (PMC12709581; doi:10.1021/acsabm.5c01890)

checkCIF/PLATON report

Structure factors have been supplied for datablock(s) EC1

THIS REPORT IS FOR GUIDANCE ONLY. IF USED AS PART OF A REVIEW PROCEDURE FOR PUBLICATION, IT SHOULD NOT REPLACE THE EXPERTISE OF AN EXPERIENCED CRYSTALLOGRAPHIC REFEREE.

No syntax errors found. CIF dictionary Interpreting this report

Datablock: EC1

Bond precision: Mg- O = 0.0018 A Wavelength=1.54178

Cell:	a=13.5126(4)	b=16.5209(5)	c=10.3783(5)
	alpha=90	beta=119.667(1)	gamma=90
Temperature:	298 K		

	Calculated	Reported
Volume	2013.15(13)	2013.15(13)
Space group	C 2/c	C2/c
Hall group	-C 2yc	-C 2yc
Moiety formula	C H14 Cl2 Mg2 O13 P2, 6(H2 O)	C H14 Cl2 Mg2 O13 P2, 6(H2 O)
Sum formula	C H26 Cl2 Mg2 O19 P2	C H26 Cl2 Mg2 O19 P2
Mr	523.68	523.68
Dx,g cm-3	1.728	1.728
Z	4	4
Mu (mm-1)	5.830	5.830
F000	1088.0	1088.0
F000'	1098.38	
h,k,lmax	16,19,12	16,19,12
Nref	1799	1785
Tmin,Tmax	0.522,0.558	0.494,0.753
Tmin'	0.473	

Correction method= # Reported T Limits: Tmin=0.494 Tmax=0.753
AbsCorr = MULTI-SCAN

Data completeness= 0.992 Theta(max)= 66.820

R(reflections)= 0.0329(1687) wR2(reflections)= 0.0976(1785)

S = 1.103 Npar= 128

The following ALERTS were generated. Each ALERT has the format
test-name_ALERT_alert-type_alert-level.
Click on the hyperlinks for more details of the test.

Alert level C

```
PLAT260_ALERT_2_C Large Average Ueq of Residue Including       O10      0.125 Check
PLAT911_ALERT_3_C Missing FCF Refl Between Thmin & STh/L=    0.596         13 Report
               14  0  0,   3  3  0,  11 11  0,  -7 17  2,  -3  3  3,   6 14  4,
               -6 18  4, -14  0 10, -13  1 11, -11  7 11, -10  8 11, -10  0 12,
               -8  0 12,
PLAT975_ALERT_2_C Check Calcd Resid. Dens.  0.99Ang From O10     .       0.52 eA-3
PLAT975_ALERT_2_C Check Calcd Resid. Dens.  1.06Ang From O9      .       0.52 eA-3
PLAT976_ALERT_2_C Check Calcd Resid. Dens.  1.05Ang From O6      .      -0.45 eA-3
PLAT977_ALERT_2_C Check Negative Difference Density on H6B       .      -0.35 eA-3
PLAT977_ALERT_2_C Check Negative Difference Density on H11A      .      -0.35 eA-3
```

Alert level G

```
PLAT002_ALERT_2_G Number of Distance or Angle Restraints on AtSite          3 Note
PLAT007_ALERT_5_G Number of Unrefined Donor-H Atoms .............          15 Report
              H4A   H4B   H5A   H5B   H6A   H6B   H7    H8A   H8B   H9A   H9B
              H10A  H10B  H11A  H11B
PLAT128_ALERT_4_G Alternate Setting for Input Space Group     C2/c       I2/a Note
PLAT176_ALERT_4_G The CIF-Embedded .res File Contains SADI Records          1 Report
PLAT232_ALERT_2_G Hirshfeld Test Diff (M-X)  Mg1      --O5       .        6.9 s.u.
PLAT232_ALERT_2_G Hirshfeld Test Diff (M-X)  Mg1      --O6       .        5.1 s.u.
PLAT300_ALERT_4_G Atom Site Occupancy of O9         Constrained at        0.5 Check
PLAT300_ALERT_4_G Atom Site Occupancy of H9A        Constrained at        0.5 Check
PLAT300_ALERT_4_G Atom Site Occupancy of H9B        Constrained at        0.5 Check
PLAT300_ALERT_4_G Atom Site Occupancy of H10A       Constrained at        0.5 Check
PLAT300_ALERT_4_G Atom Site Occupancy of H10B       Constrained at        0.5 Check
PLAT302_ALERT_4_G Anion/Solvent/Minor-Residue Disorder (Resd    3)       100% Note
PLAT303_ALERT_2_G Full Occupancy Atom H5A       with # Connections       2.00 Check
PLAT303_ALERT_2_G Full Occupancy Atom H5B       with # Connections       2.00 Check
PLAT303_ALERT_2_G Full Occupancy Atom H6A       with # Connections       2.00 Check
PLAT303_ALERT_2_G Full Occupancy Atom H6B       with # Connections       2.00 Check
PLAT304_ALERT_4_G Non-Integer Number of Atoms in ..... (Resd    3)       1.50 Check
PLAT417_ALERT_2_G Short Inter D-H..H-D       H6B      ..H10A     .       1.98 Ang.
                                                  2-x,y,3/2-z  =      2_756 Check
PLAT779_ALERT_4_G Suspect or Irrelevant (Bond) Angle(s) in CIF ...       0.00 Deg.
              H9A  -O9   -H9A     1_555   1_555   1_555 ......     #   49 Check
PLAT779_ALERT_4_G Suspect or Irrelevant (Bond) Angle(s) in CIF ...       0.00 Deg.
              H9B  -O9   -H9B     1_555   1_555   1_555 ......     #   54 Check
PLAT789_ALERT_4_G Atoms with Negative _atom_site_disorder_group  #          3 Check
PLAT822_ALERT_4_G CIF-embedded .res Contains Negative PART Numbers          1 Check
PLAT860_ALERT_3_G Number of Least-Squares Restraints ............           1 Note
PLAT883_ALERT_1_G No Info/Value for _atom_sites_solution_primary .     Please Do !
PLAT909_ALERT_3_G Percentage of I>2sig(I) Data at Theta(Max) Still        90% Note
PLAT910_ALERT_3_G Missing # of FCF Reflection(s) Below Theta(Min).          1 Note
                1  1  0,
PLAT913_ALERT_3_G Missing # of Very Strong Reflections in FCF ....          1 Note
               -3  3  3,
PLAT941_ALERT_3_G Average HKL Measurement Multiplicity ..........         4.6 Low
PLAT969_ALERT_5_G The 'Henn et al.' R-Factor-gap value ..........        3.31 Note
              Predicted wR2: Based on SigI**2  2.95 or SHELX Weight  9.18
```

```
  0 ALERT level A = Most likely a serious problem - resolve or explain
  0 ALERT level B = A potentially serious problem, consider carefully
  7 ALERT level C = Check. Ensure it is not caused by an omission or oversight
 29 ALERT level G = General information/check it is not something unexpected

  1 ALERT type 1 CIF construction/syntax error, inconsistent or missing data
 14 ALERT type 2 Indicator that the structure model may be wrong or deficient
  6 ALERT type 3 Indicator that the structure quality may be low
 13 ALERT type 4 Improvement, methodology, query or suggestion
  2 ALERT type 5 Informative message, check
```

It is advisable to attempt to resolve as many as possible of the alerts in all categories. Often the minor alerts point to easily fixed oversights, errors and omissions in your CIF or refinement strategy, so attention to these fine details can be worthwhile. In order to resolve some of the more serious problems it may be necessary to carry out additional measurements or structure refinements. However, the purpose of your study may justify the reported deviations and the more serious of these should normally be commented upon in the discussion or experimental section of a paper or in the "special_details" fields of the CIF. checkCIF was carefully designed to identify outliers and unusual parameters, but every test has its limitations and alerts that are not important in a particular case may appear. Conversely, the absence of alerts does not guarantee there are no aspects of the results needing attention. It is up to the individual to critically assess their own results and, if necessary, seek expert advice.

Publication of your CIF in IUCr journals

A basic structural check has been run on your CIF. These basic checks will be run on all CIFs submitted for publication in IUCr journals (*Acta Crystallographica*, *Journal of Applied Crystallography*, *Journal of Synchrotron Radiation*); however, if you intend to submit to *Acta Crystallographica Section C* or *E* or *IUCrData*, you should make sure that full publication checks are run on the final version of your CIF prior to submission.

Publication of your CIF in other journals

Please refer to the *Notes for Authors* of the relevant journal for any special instructions relating to CIF submission.

PLATON version of 06/01/2024; check.def file version of 05/01/2024

Datablock EC1 - ellipsoid plot

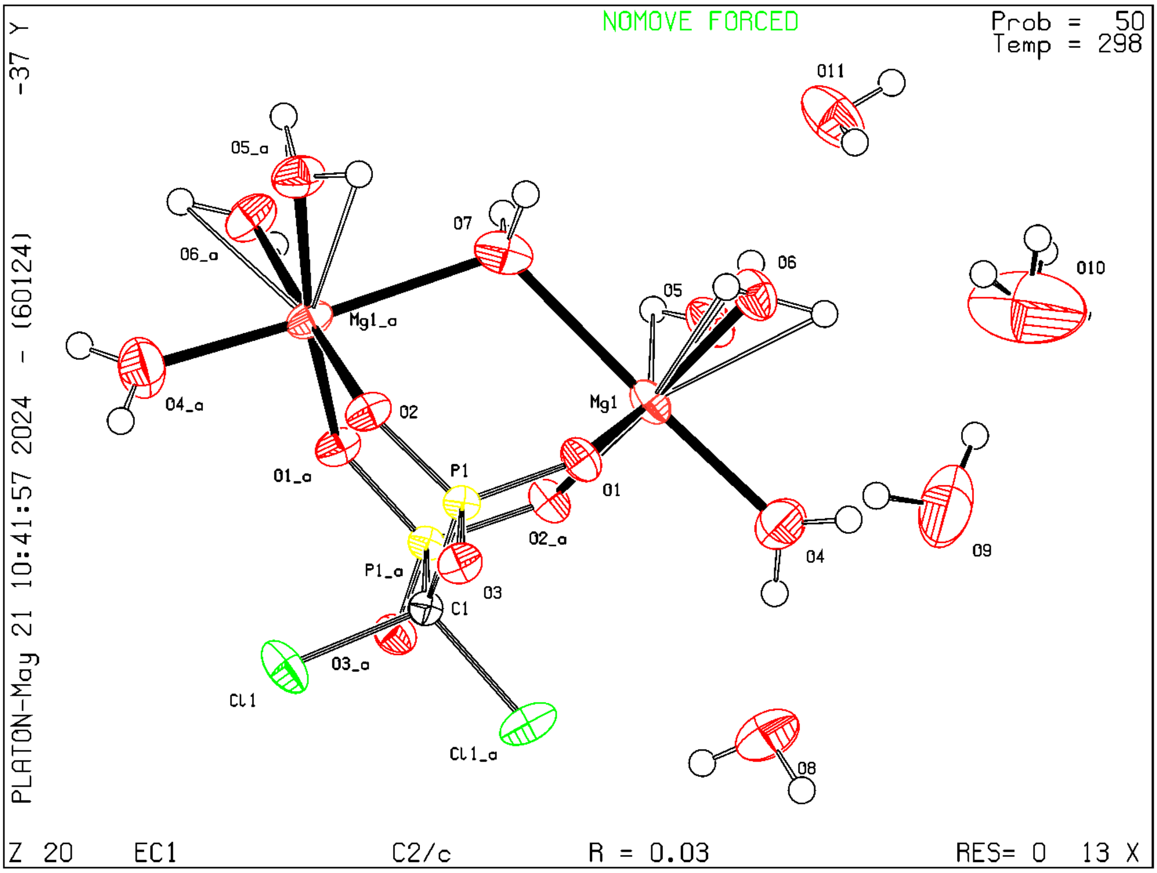

Supplement: Supplementary file 5 [file mt5c01890_si_005.pdf]
